# Diversity of Useful Plants in Cabo Verde Islands: A Biogeographic and Conservation Perspective

**DOI:** 10.3390/plants11101313

**Published:** 2022-05-15

**Authors:** Maria Cristina Duarte, Isildo Gomes, Silvia Catarino, Miguel Brilhante, Samuel Gomes, Aline Rendall, Ângela Moreno, Arlindo Rodrigues Fortes, Vladmir Silves Ferreira, Isaurinda Baptista, Herculano Dinis, Maria Manuel Romeiras

**Affiliations:** 1 Centre for Ecology, Evolution and Environmental Changes (cE3c) & Global Change and Sustainability Institute (CHANGE), Faculdade de Ciências, Universidade de Lisboa, 1749-016 Lisbon, Portugal; mmromeiras@isa.ulisboa.pt; 2 Instituto Nacional de Investigação e Desenvolvimento Agrário (INIDA), São Jorge dos Órgãos, Praia CP 84, Cape Verde; isildo.gomes@inida.gov.cv (I.G.); samuel.gomes@inida.gov.cv (S.G.); aline.rendall@inida.gov.cv (A.R.); aveigamoreno@gmail.com (Â.M.); 3 Linking Landscape, Environment, Agriculture and Food (LEAF) Research Center & Associated Laboratory TERRA, Instituto Superior de Agronomia (ISA), Universidade de Lisboa, Tapada da Ajuda, 1349-017 Lisbon, Portugal; scatarino@isa.ulisboa.pt (S.C.); miguelbrilhante131@hotmail.com (M.B.); 4 Forest Research Center (CEF), Instituto Superior de Agronomia (ISA), Universidade de Lisboa, Tapada da Ajuda, 1349-017 Lisbon, Portugal; 5 Escola Superior de Ciências Agrárias e Ambientais, Universidade de Cabo Verde, Praia CP 84, Cape Verde; arlindo.fortes@docente.unicv.edu.cv (A.R.F.); vladmir.ferreira@adm.unicv.edu.cv (V.S.F.); isaurinda.baptista@adm.unicv.edu.cv (I.B.); 6 Centre for African and Development Studies (CESA), Lisbon School of Economics and Management, Universidade de Lisboa, 1249-078 Lisbon, Portugal; 7 Associação Projecto Vitó, Xaguate, Sao Filipe CP 47, Cape Verde; herculanodinis86@hotmail.com

**Keywords:** food security, historical perspective, Macaronesia islands, native plants, sustainable exploitation of natural resources, traditional uses

## Abstract

Cabo Verde’s biodiversity is threatened by activities that meet human needs. To counteract this, an integration of scientific and indigenous knowledge is required, but no comprehensive list of the useful local plants is available. Thus, in this work, we assess (1) their diversity and phytogeography; (2) the role of geophysical, historical, and socio-economic factors on species distribution and uses; and (3) potentially relevant species for sustainable development. Data were obtained from flora, scientific publications, historical documents, herbarium specimens and field work. Many species were introduced since the 15th century to support settlement and commercial interests. We identified 518 useful taxa, of which 145 are native, 38 endemic and 44 endangered. The number of useful taxa is correlated with altitude and agricultural area, as well as with rural population indicators, but not with total population or socio-economic indicators such as gross domestic product. Native taxa are mostly used for fuelwood, forage and utilitarian purposes. Agrobiodiversity and traditional practices seem crucial to cope with recurrent droughts and ensure food security. Most of the introduced species do not present conservation problems, contrasting with the overuse of some native taxa. The safeguarding of native populations will ensure the sustainable exploitation of these resources and benefit the local economy.

## 1. Introduction

A sixth mass extinction of life on Earth is under way, and habitat loss is among the most important anthropogenic threats, followed by over-exploitation, introduced species and climate change, leading to a loss of species and ecosystems [1]. Although island and mainland regions have undergone equivalent past habitat loss, projections indicate that land-use-driven changes to islands might be stronger in the future. Given their conservation risks, smaller land areas and high levels of endemism richness, islands may offer particularly high returns for species conservation efforts and therefore warrant a high priority in global biodiversity conservation [2].

In the north-eastern Atlantic Ocean, the Macaronesian archipelagos (i.e., Azores, Madeira, Selvagens, Canary Islands and Cabo Verde) are an outstanding center of biodiversity but also one of the most threatened areas, mainly by human activity. These islands show a strong climatic gradient from an oceanic temperate climate in the Azores to a warm arid climate in Cabo Verde [3]. Cabo Verde is vulnerable to natural disasters, and particularly rural populations are dependent on natural resources and on agriculture-based economy [4,5]. Therefore, the need to integrate local indigenous knowledge for sustainable management and conservation of natural resources is increasingly recognized. Recently, some studies have focused on important plant families widely used as food and forage sources (e.g., pulses (Fabaceae) [6] and millets (Poaceae) [7]). In addition, the possible economic benefits, especially from medicinal species [8] or native forest products [9], have been emphasized as particularly attractive approaches for economically weak countries such as Cabo Verde. However, there is limited knowledge of useful species in this and other Macaronesian archipelagos, and only a few complementary initiatives have been undertaken, such as the Spanish network of autochthonous plant genetic resources and wild plant (Red Española de Bancos de Germoplasma de Plantas Silvestres y Fitorrecursos Autóctonos, REDBAG).

The use of plant species is a common ancestral practice and has been an invaluable resource since the colonization of Cabo Verde islands [10]. Whether native or introduced, several species are particularly important as sources of food, forage, medicines, building materials, fiber and fuel, among others, especially for local communities [11], providing not only economic income, particularly relevant in natural resource-poor regions, as is the case of Cabo Verde, but also playing important social and cultural roles for local people. The progressive abandonment of centuries-old uses and practices, together with land use shifts, particularly the extensive forestation [12,13] or the development of tourism [14], justifies an urgent assessment of the plants traditionally used in Cabo Verde. Previous works (e.g., [11,15,16,17]) pinpoint the importance of such resources for population subsistence and wellbeing. However, knowledge about such plants is incomplete, and no exhaustive approach has been conducted so far.

To enhance the livelihoods of local communities, and in line with global efforts (e.g., Aichi Biodiversity Target 18, concerning the safeguarding of the traditional knowledge), we aimed to gather the available traditional knowledge and practices about useful plants from Cabo Verde, using an integrative approach (i.e., biological, ecological and historical), to provide crucial data not only with scientific purposes but also for local government policies with respect to agriculture and the conservation of plant genetic resources. This information is essential to assess the socio-economic value of the archipelago’s flora as a provider of widely diverse ecological services such as supplying food and other goods for human use, forage to feed livestock and control of soil erosion, while serving as a basis to assess the potential risks of these uses to their sustainability and conservation in Cabo Verde.

Thus, this work aims to (1) assess the taxonomic diversity and phytogeographic distribution of the useful plants in Cabo Verde; (2) establish the role of geophysical characteristics of the islands, as well as of the demographic, historical, economic and socio-cultural factors driving the distribution patterns of the species and their uses; and (3) identify relevant species, particularly native ones, with a future relevant role in the sustainable development of the archipelago.

## 2. Results

### 2.1. The First Reported Useful Species—A Brief Historical Note

Cabo Verde was uninhabited until 1456, when it was discovered by the Portuguese, and the species introduced by the settlers can be retrieved from the known historical documents. Sugarcane, figs, grapes and melons, among other fruits largely used in mainland Portugal, are referenced as early as 1506 by Valentim Fernandes (in Monod et al. [18]). By 1545, the accounts of Piloto Anónimo (in Sauvageot [19]) include citruses (such as oranges, lemons and citrons), pomegranates, coconuts and vegetables. By the end of the 16th century, Gaspar Frutuoso (in Frutuoso [20]) also mentions cotton, bananas, pears, beans, pumpkins and “Milho branco e grado de maçaroca e milho miúdo”, probably referring to small grain millets and sorghum [21] used in West Africa. Already in the 18th century, the British sailor George Roberts, who stayed for two years (1722–1724) in the archipelago, reported maize (*Zea mays*) and cassava (*Manihot esculenta*), two American crops introduced into Africa in the 16th century, as well as beans, guinea millets (possibly referring to species of the genus *Urocholoa*), pumpkins (*Cucurbita pepo*), fruit trees such as lemons (*Citrus × limon*), sweet and sour oranges (*Citrus × aurantium*), limes (*Citrus × aurantiifolia*), cidron (*Citrus medica*), guavas (*Psidium* spp.), sugar-apples (*Annona* spp.), tamarinds (*Tamarindus indica*), coconuts (*Cocos nucifera*), pineapples (*Ananas comosus*), plantains (*Musa* spp.), sweet potatoes (*Ipomoea batatas*), watermelons (*Citrullus lanatus*) and melons (*Cucumis melo*) [22]. In addition, cotton (*Gossypium* spp.) and indigo (*Indigofera tinctoria*) were mainly reported from Boavista, Maio and Santo Antão, as well as sugarcane plantations (*Saccharum officinarum*) and vineyards (*Vitis vinifera*) in Santiago, Fogo and São Nicolau.

In 1772, the botanist Johann Forster was in Santiago, and in his short list of collected species, most of them already mentioned by George Roberts, he included several tropical American species, such as papaya (*Carica papaya*), *Vachellia farnesiana* and *Caesalpinia pulcherrima*, a beautiful ornamental tree, and Asian basil (*Ocimum basilicum*) [23].

By the end of the 18th century, João da Silva Feijó, a Portuguese naturalist taking part in the “Philosophical Journeys” under the patronage of the Portuguese Crown, made an extensive work in Cabo Verde and provided the inventory of natural productions of the islands. Between 1783 and 1789, he collected hundreds of species, reported in his manuscripts (e.g., in Feijó [24]; for details see Gardère [25]). The lists, including both native and exotic species, show that more than 50 economically relevant species from all over the world were already established in the archipelago by then. Adding to those reported by previous explorers, species are mentioned such as the African *Adansonia digitata*, *Coffea arabica*, *Hibiscus sabdariffa*, and *Ricinus communis*; the American *Annona squamosa*, *Arachis hypogaea*, *Capsicum frutescens*, *Furcraea foetida, Gossypium hirsutum*, *Jatropha curcas*, *Mammea americana*, *Nicotiana tabacum, Opuntia ficus-indica*, *Physalis peruviana*, and *Spondias mombin*; the Asiatic *Abelmoschus esculentus*, *Cassia fistula* and *Rhaphiolepis bibas*, or the European *Ceratonia siliqua*, *Cydonia oblonga*, *Malus domestica*, *Ficus carica* and *Pyrus communis*, to mention only some examples.

The list of exotic species with economic interest present in Cabo Verde did not cease to grow in the following centuries (e.g., [26,27,28]), and most of them still occur in the archipelago. The introduction of new food species is continuous, as shown with the recent introduction of the American dragon fruit (epiphytic cacti of the genus *Selenicereus*) or the star fruit (*Averrhoa carambola*).

In Cabo Verde, the association of different crops is a common practice. Already indicated in historical texts, this may be related to the restricted availability of suitable land for agricultural activities (e.g., watered valleys, well-exposed slopes), leading to the concentration of a wide diversity of crops in small areas. Currently, these practices are one of the ways in which farmers minimize the risks both of pests and diseases and of climatic irregularity, seeking to ensure the success of at least some of the productions. Thus, it is common to find maize, cabbage (*Brassica oleracea*), potatoes, pumpkins (*Cucurbita* spp.), beans and fruit trees such as bananas (*Musa* spp.), avocado (*Persea americana*), guava (*Psidium guajava*) and lemon as well as sugarcane, tomato (*Solanum lycopersicum*), carrot (*Daucus carota*), papaya and mango (*Mangifera indica*) [29] growing together in small fields.

### 2.2. Taxonomic Diversity

The complete lists of plants used in Cabo Verde, with common names and respective uses, are presented in Table 1 (endemic and non-endemic native taxa) and in Table 2 (introduced taxa); species for which there is information on their historical use (until the end of the 18th century) are also indicated.

**Figure 1:**
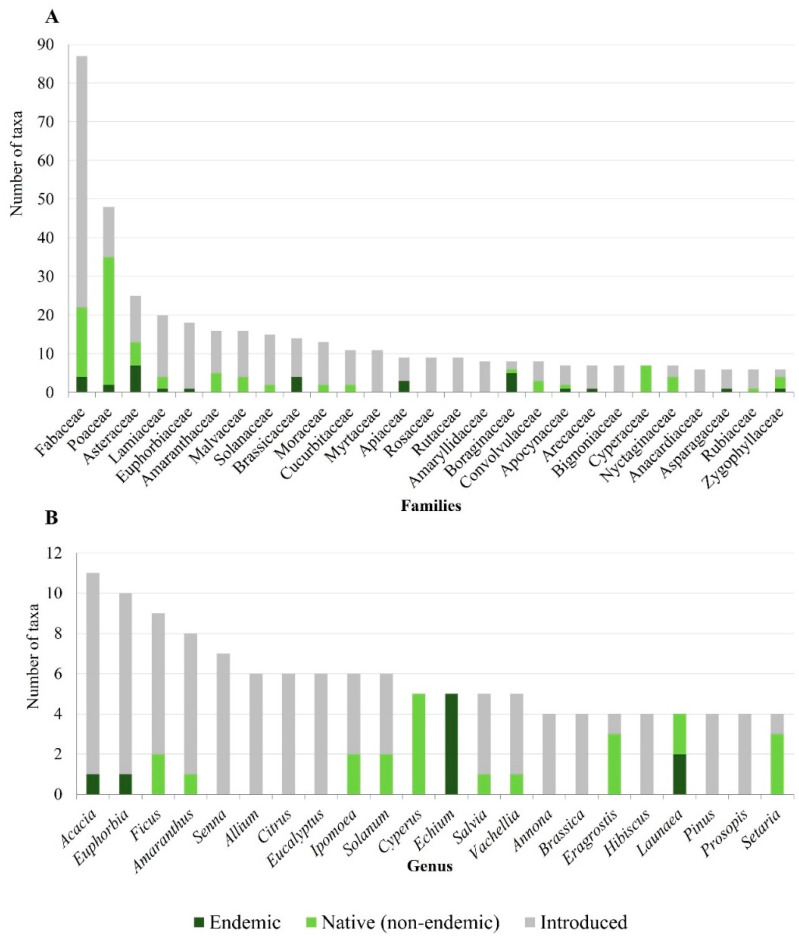
Number of endemics, native non-endemics and introduced useful taxa identified in Cabo Verde. (**A**) Family (only families with more than five taxa are represented); (**B**) Genus (only genera with more than three taxa are represented).

We identified 518 taxa belonging to 338 genera and 88 families (Supplementary Materials Table S1). The best represented families are the Fabaceae, with 87 taxa (four endemics); the Poaceae, with 48 taxa (two endemics); the Asteraceae, with 25 taxa (seven endemics); and the Lamiaceae, with 20 taxa (one endemic) (Figure 1A). With 11 taxa*, Acacia* is the most diverse genus, followed by *Euphorbia* (10), *Ficus* (9), *Amaranthus* (8) and *Senna* (7) (Figure 1B).

Most of the useful plants found in Cabo Verde were introduced (373 taxa, representing 72%) (Supplementary Materials Table S1); among them, about 86% are cultivated in the country. Except for the Poaceae, most of the better-represented families are mainly composed of exotic species (Figure 1A). Exotics also predominate in the best-represented genera (e.g., *Acacia*, *Euphorbia*, *Ficus* or *Amaranthus*), with the exceptions of *Cyperus* (Cyperaceae), *Echium* (Boraginaceae), *Launaea* (Asteraceae) and *Eragrostis* and *Setaria* (Poaceae) (Figure 1B).

There are 145 native taxa, 38 of them endemic, with Asteraceae (7), Boraginaceae (5), Fabaceae (4), Brassicaceae (4), Apiaceae (3) and Poaceae (2) contributing the largest number of endemic species.

**Figure 2:**
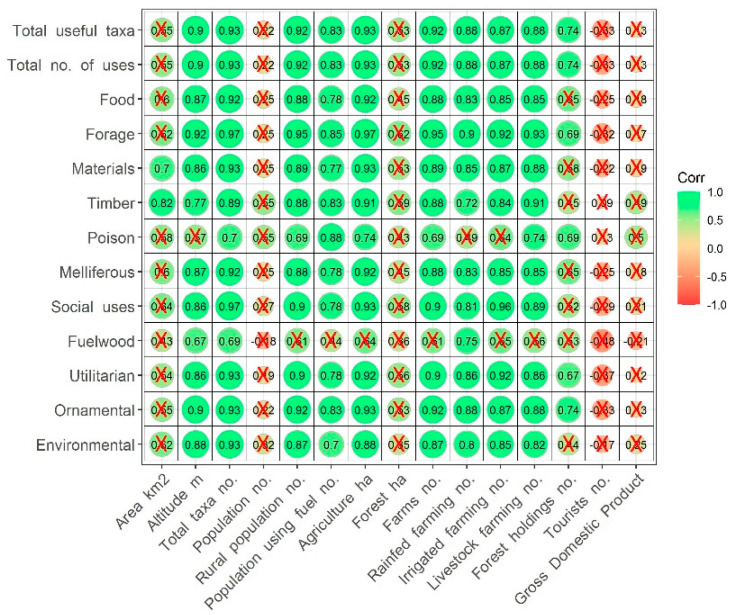
Spearman correlation coefficients between useful taxa (total number and classes of use) and geographic, demographic and economic indicators. Color-coded correlation scale is provided on the right of the plot (green represents positive correlations, and red represents negative correlations); darker color tones and larger circles represent larger correlation coefficients. Values marked with an X are not statistically significant (*p*-value > 0.05).

Considering the distribution of the useful species in the nine Cabo Verde islands (Santa Luzia, an uninhabited island, is not included in this analysis), there are very high positive correlations of “total useful taxa” with “altitude” (r = 0.900), area occupied by agriculture (r = 0.933), as well as with “total taxa number” (useful or not) present in each island (r = 0.933) (Figure 2). The “rural population” and the “total number of farms” display high positive correlations as well as all the farm categories: “rainfed”, “irrigated”, and “livestock farming”. Less relevant (moderately positive) is the correlation with “forest holdings”.

No significant correlations were found between the “total useful taxa” and the variables “total population”, and indicators of other economic activities such as “tourists” or “gross domestic product” (GDP).

### 2.3. Main Uses of Cabo Verdean Flora

**Figure 3:**
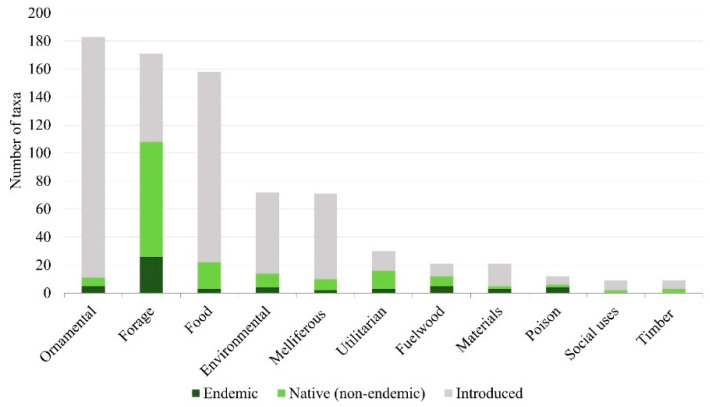
Main uses of the useful taxa found in Cabo Verde and respective origin in Cabo Verde.

Among the 11 considered classes of use, the most frequent are ornamental, with 183 taxa (corresponding to 35.3%); forage and pasture, with 171 taxa (33%); food, with 158 taxa (30.5%); environmental, with 72 taxa (13.9%); and melliferous with 71 (13.7%). The other classes represent less than 10% each (Supplementary Materials Table S1 and Figure 3).

Some taxa are used for multiple purposes, for instance, *Vachellia nilotica* subsp. *indica* is used for ornamental purposes and for forage, fuelwood, materials, and environmental objectives, in addition to being a melliferous plant. *Moringa oleifera* is a very useful plant for alimentary, pasture, ornamental and materials purposes. In addition, the native *Ziziphus mauritiana* is used for food, forage, melliferous, fuelwood and timber. However, most taxa (350 taxa, corresponding to 67.6%) are reported for only one use, the top three being ornamental (111), forage (89) and food (88).

Native plants (including endemic taxa) represent most of the taxa used as fuelwood (57.1%), as forage (63.2%) and for utilitarian applications (53.3%) (Figure 3). Most of the endemic taxa (26) are reported as forage, exceeding the other categories by far. Exotic species are mainly present in the other categories and are particularly well-represented as ornamentals (172 taxa, 94%).

### 2.4. Growth form Diversity and Uses

**Figure 4:**
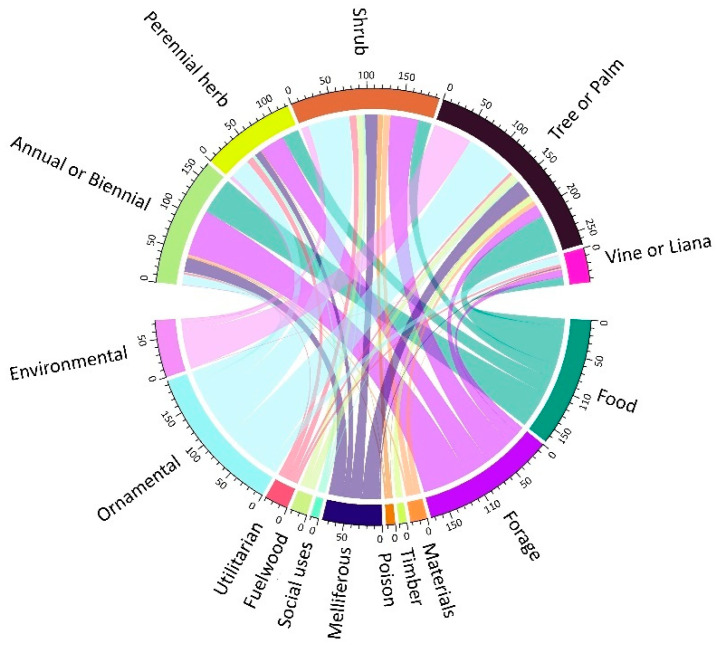
The chord diagram shows the relation between the uses and the habit of the taxa. The areas are proportional to the number of taxa.

The useful plants of Cabo Verde present a high diversity of growing habits and life cycles. About 28.4% are trees or palms, 25.3% are shrubs or subshrubs, 23.2% are annual or biennial herbs, 16.8% are perennial herbs and 6.3% are climbers, including vines and lianas (Figure 4). Trees are the most used for environmental purposes and, as expected, timber; annuals and biennials are most commonly used for forage and human food; ornamentals are mostly trees and shrubs.

The use categories encompassing the widest variety of species are forage, human food and ornamental, including all growth forms.

### 2.5. Distribution across Cabo Verde Archipelago

**Figure 5:**
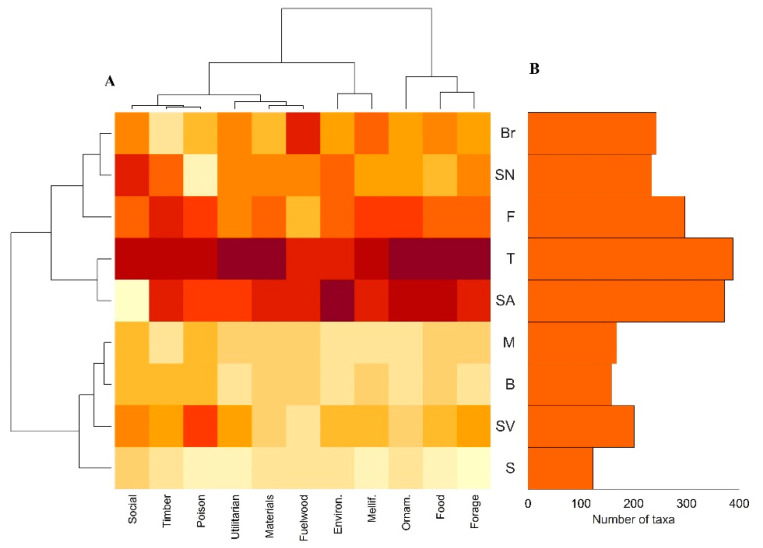
(**A**) Heatmap of the categories of uses identified for the species occurring in each island. Number of records of useful plants per category of use (axis *x*) and islands (*y* axis). Yellow boxes indicate the low values, and dark red boxes indicate high values. The heatmap was constructed based on a correlation matrix; the horizontal lines are the clusters of Cabo Verde islands (B—Boavista; Br—Brava; F—Fogo; M—Maio; S—Sal; T—Santiago; SA—Santo Antão; SN—São Nicolau; SV—São Vicente) and the vertical columns are the clusters of the use categories. (**B**) Number of useful taxa per island.

Santiago and Santo Antão are the islands with more useful taxa, at 388 and 372 taxa, respectively (Supplementary Materials Table S1, Figure 5B). Fogo, Brava and São Nicolau have 297, 243 and 234 taxa, respectively. The remaining islands have between 203 (São Vicente) and 123 taxa (Sal).

The islands where agricultural activities prevail (i.e., with higher ”number of farms”, or larger “agriculture area”—Supplementary Materials Table S2), are also those where higher numbers of useful taxa are reported (e.g., Santo Antão, Santiago and Fogo).

Based on the number of species per use category (see heatmap in Figure 5A), the UPGMA analysis reveals two main groups of islands: (1) Santo Antão, Santiago, Fogo, São Nicolau and Brava, the islands with the highest values for most of the use categories, with Santiago presenting the highest values for ten categories; and (2) a group including the remaining islands, with the lowest values in most of the use categories.

Correlation coefficients of “total number of uses” and individual uses roughly follow the same pattern as the “total useful taxa” (Figure 2).

### 2.6. Uses vs. Biogeographic Origin

The huge biogeographic diversity of exotic species among the useful flora of Cabo Verde is remarkable.

**Figure 6:**
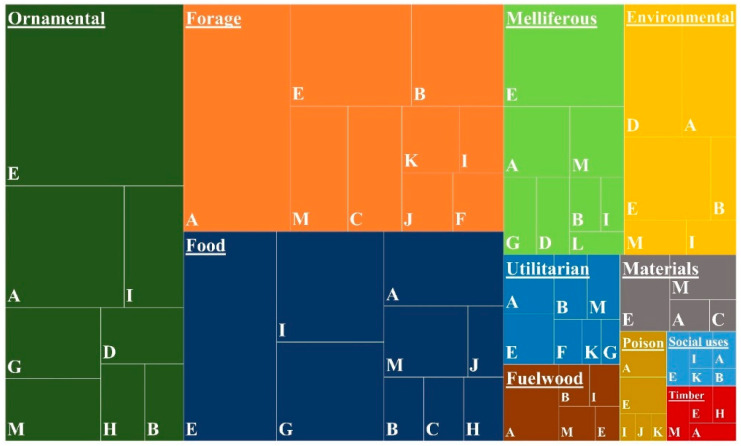
Relationship between taxa uses and biogeographic origin. The areas of the polygons are proportional to the number of taxa. (**A**): Afrotropical; (**B**): Afrotropical and Oriental; (**C**): Afrotropical, Oriental and Austral (optional); (**D**): Austral; (**E**): Neotropical; (**F**): Neotropical, Afrotropical, Oriental and Austral (optional); (**G**): Oriental; (**H**): Oriental and Austral; (**I**): Palaearctic; (**J**): Palaearctic and Afrotropical; (**K**): Palaearctic, Afrotropical, Oriental, and Austral (optional); (**L**): Nearctic; (**M**): Other. Notes: Afrotropical (includes Afrotemperate region); Austral (includes one or more of the Neoguinean, Australotemperate and Neozelandic regions); Neotropical (includes Andean region).

Taxa of Afrotropical origin prevail as forage (Figure 6). That is the case for grass species such as *Andropogon gayanus* and *Urochloa xantholeuca*, reported as excellent forage, or the leguminous species from the genera *Crotalaria*, *Desmodium*, *Grona*, *Macrotyloma*, *Rhynchosia*, *Sesbania*, *Tephrosia* and *Vigna*, besides several endemic species of *Lotus*.

The taxa used for ornamental purposes are mainly of Neotropical origin, namely Apocynaceae (e.g., *Asclepias curassavica*, *Cascabela thevetia*, *Plumeria rubra*), Fabaceae (e.g., *Caesalpinia pulcherrima*), and Lamiaceae (e.g., *Salvia* spp.), only to mention a few examples. Of the same origin are most taxa used as food (e.g., species of the genera *Amaranthus*, *Annona*, *Cucurbita*, *Capsicum*, and *Solanum*). The Neotropical region is also the main origin of melliferous plants and of the taxa used to obtain materials and timber, however with considerably lower importance.

The Austral origin prevails in the environmental purposes category, with the well-represented genera *Acacia* and *Eucalyptus*.

Most taxa (364 taxa) occur in only one biogeographic region. Overall, 432 taxa, corresponding to 83.4% of all the useful taxa, occur as native in either Afrotropical, Neotropical, Australotropical or Oriental regions (in single or mixed classes). Of the 86 taxa that do not occur in at least one of these regions, more than half (54) have an exclusively Palearctic distribution.

### 2.7. Native Species Conservation

Only 202 (37.6%) of the studied taxa were assessed by the International Union for Conservation of Nature (IUCN) Red List of Threatened Species [36] and Romeiras et al. [37]. Most of them (144) are classified as Least Concern (LC), 7 as Near Threatened (NT), 9 as Vulnerable (VU), 23 as Endangered (EN), and 5 as Critically Endangered (CR). Fourteen taxa are classified as Data Deficient (DD).

Forage is the use category that includes most threatened Cabo Verdean species (about 20), and most of them occur in highlands communities [37]. Several species can be pointed out as examples: *Diplotaxis glauca* (CR), *Tornabenea tenuissima* (CR), *Conyza feae* (EN), *Echium stenosiphon* (EN), *Echium vulcanorum* (EN), *Globularia amygdalifolia* (EN), *Helianthemum gorgoneum* (EN), *Tornabenea annua* (EN), *Tornabenea bischoffii* (EN), *Periploca chevalieri* (EN), *Sonchus daltonii* (EN), *Launaea picridioides* (VU), *Forsskaolea procridifolia* (NT) and *Lavandula rotundifolia* (NT). Besides these herbaceous or shrub species, also trees, such as *Phoenix atlantica* (EN), are reported as fodder (leaves). Classified as Data Deficient, several species of *Lotus* (e.g., *L. brunneri*, *L. jacobaeus*, and *L. purpureus*) are also well known for their major importance as forage.

Two Endangered endemic species are used for their edible fruits: *Phoenix atlantica* and *Sideroxylon marginatum*; and *Urochloa caboverdiana*, classified as Vulnerable, is used in times of food shortage (seeds).

The populations of several endemics, such as *Echium vulcanorum*, *E. hypertropicum* (EN), *Euphorbia tuckeyana* (NT) and *Sideroxylon marginatum*, have been depleted for firewood or charcoal. Also threatened are species once widely used for leather tanning, such as *Periploca chevalieri* (leaves), and *Euphorbia tuckeyana* (sap). *Asteriscus daltonii* subsp. *vogelii* (NT) and *Pulicaria diffusa* (EN) are reported as used in fumigations, and *Dracaena caboverdeana* (CR), produces the famous dragon’s blood, a red resin used as varnish, besides being a valuable ornamental species. Other relevant endemic ornamentals are the palm *Phoenix atlantica* and the Crassulaceae *Aeonium gorgoneum* (EN).

The use of some non-endemic native taxa is also of concern. This is the case with the use as fuelwood of *Arthrocaulon franzii*, used in lime kilns, *Tamarix senegalensis*, and *Tetraena gaetula* subsp. *waterlotii*.

Some of the introduced species are also classified as threatened in their native areas of distribution (e.g., *Jacaranda mimosifolia*, *Kalanchoe daigremontiana*, or *Khaya senegalensis*). However, in most cases, the unknown origin of the introduced plants in Cabo Verde (wild populations or plant nurseries) hampers a correct evaluation of their relevance for species conservation.

### 2.8. Agrobiodiversity and Traditional Knowledge

The plants cultivated and preserved by rural communities for a long time and, as such, extremely well adapted to the diversity of bioclimatic conditions of the archipelago constitute a valuable reservoir of plant genetic resources. The cultivation of this agrobiodiversity, together with the traditional knowledge on cultural practices (selection, propagation, and conservation), is crucial to face the drought cycles that are common in Cabo Verde and to ensure food security. However, in drought years, many of these genetic resources are lost, as farmers lose their seeds when crops fail to grow due to lack of rain.

Beans are perhaps the crop with most varieties. For example, in Santo Antão, the “feijão-caqui” (a variety of *Lablab purpureus* subsp. *purpureus*), highly resistant to dryness, keeps the pods closed at the end of maturation, thus avoiding the need to collect grains from the ground [29]. Regarding corn, the selection is made by choosing the best ears (those fully filled with grains and with more “rows”), which are not threshed until the time of sowing and from which only the largest and best-formed grains are used [29].

An example of a traditional technique for propagule conservation is, in Santo Antão, the storage of potatoes (*Solanum tuberosum*) in cool places such as caves, dug out of pozzolanic rocks, which are very common in the region due to their volcanic origin [29].

In Santiago, it is documented that sweet potato (*Ipomoea batatas*) seedlings or cuttings are sold/offered to farmers in highlands, where the cooler climate allows for their conservation; during the planting season, these same plants are again sold/offered to farmers in lower areas. This is a secular practice that is used in this and also in other islands (e.g., Fogo) and also with other crops such as cassava.

To prevent the emergence of pests during storage of seeds and the serious damage they cause, namely in maize and beans, plant species that are rich in essential oils and act as biocides are traditionally used. This is the case of pink pepper leaves (*Schinus molle*), “losna” (*Artemisia gorgonum*), neem (*Azadirachta indica*), laurel (*Laurus nobilis*), leaves and fruits of eucalyptus (*Eucalyptus globulus*) and fruits of chilli pepper (*Capsicum frutescens*). The latter seem to allow the conservation of seeds and maintain their germination ability for many years [29].

## 3. Discussion

In Cabo Verde, as well as worldwide, the use of plants for a variety of purposes is a common practice. The data provided in this paper improve our knowledge of the flora used by local populations in Cabo Verde and underline the high dependence of the populations on the use of plants for multiple purposes.

Our comprehensive inventory, including historical works, collected previously ignored information on particular uses of species and also drew attention to some species that are no longer used, contributing to the general knowledge of useful species, as has been done in other tropical regions, such as South America (e.g., Cámara-Leret et al. [38]), Asia (e.g., Vu and Nguyen [39]) or Africa (e.g., Nortje and van Wyk [40]; Welcome and Van Wyk [41]), where this knowledge is still insufficient.

### 3.1. Taxonomic Diversity

Useful plant species make a considerable portion of total Cabo Verdean flora. With 518 taxa, they are mainly represented by introduced species (72%).

Species not previously recorded for the archipelago, to the best of our knowledge, are here reported, e.g., *Tabebuia rosea* (a Bignoniaceae with several centuries-old specimens and recently used as ornamental street tree) and the Fabaceae *Gliricidia sepium*, both in Santiago Island.

Following a common worldwide pattern [42], the three most diverse families of useful plants are the Fabaceae, the Poaceae and the Asteraceae, with a high number of crops of global significance. In addition, these families are among the largest plant families, corresponding, respectively, to the first (Asteraceae), third (Fabaceae), and fifth (Poaceae) best-represented families [43].

The best represented families are mostly composed of exotic species, with Poaceae being an exception (see Figure 1A). The high dispersal ability as well as its extraordinary adaptability to dryness [44] place this family among the most successful in the archipelago.

Species diversity is closely related with altitude, with the highest islands (Santiago, Santo Antão and Fogo) presenting the highest numbers of useful species. Furthermore, the two groups highlighted on the heat map (see Figure 5A) show that altitude is responsible for a similar pattern in the distribution of the types of uses among islands. This is certainly related with the greater suitability of high-altitude islands, with better climatic conditions, for agricultural activities (consequently housing a larger rural population) and also to the presence of a richer flora [13,45]. Moreover, reinforcing the relevance, in this respect, of more traditional economic sectors, such as agriculture, forestry and livestock farming, the diversity of useful plants is neither related with touristic activities nor with the gross domestic product—two indicators that are highly correlated (r = 0.800; data not shown).

### 3.2. History behind Diversity

The archipelago was uninhabited until it was discovered by the Portuguese in 1456, and most of Cabo Verde’s inhabitants are of mixed Portuguese and African ancestry [46]. With a five century long settlement history, the combined influences of both cultures are evident in the use of plants, with many commonly used species (e.g., fruits and vegetables from Europe, cereals from West Africa). This knowledge was further enriched with the introduction of useful plants from other regions, such as the American continent (namely Brazil and Western Indies), resulting from the overseas trade of the Portuguese since as early as the 16th century [47].

In the early times, the introduced species were essential, if not decisive, for human survival. At the same time, they supported the dominant commercial interests linked to the slave trade and supply of merchant ships.

It is interesting to reference the example of the attempt of the first European settlers to maintain their eating habits in Cabo Verde. According to Torrão [48], seeds and other propagules accompanied the Portuguese colonizers, hoping that their Mediterranean crops would provide both food and a sociocultural link to their homeland. Early chroniclers such as Valentim Fernandes by 1506 (in Monod et al. [18]) reported that, in Santiago Island, some valleys were cultivated with fruit trees used in Europe (e.g., pears, apples, oranges, lemons, figs, grapes, etc.), reflecting the wish of European settlers to maintain their own traditions. However, the climatic constraints of the tropical climate seldom allowed the successful development of some of them (e.g., the cultivation of some cereals such as wheat and barley).

In addition, with the slaves from the West African coasts came the crops included in their dietary habits, namely rice (*Oryza glaberrima*), and “milho”, referring to *Sorghum* and/or *Pennisetum*, two common crops in Guinean coasts (not *Zea mays*, the maize from South America, not yet introduced in Africa, and later also named “milho” by the Portuguese) [48]. The emotional memory attached to food was certainly an important promoter of the plant diversity that is still found everywhere in these islands.

The high number of species whose use dates back to the beginning of colonisation is remarkable. Historical documents (until the end of the 18th century) report almost 70 species, not including vegetables, which are rarely mentioned (see Table 1 and Table 2).

Even considering the overall climatic constraints, the orographic diversity of the archipelago accounts for a wide range of habitat types, allowing the presence of species from temperate zones, such as Northern Europe or New Zealand, as well as of those with a tropical or subtropical distribution, e.g., from Central and South America or from India. In fact, introduced species have been a constant presence in Cabo Verde since the early times of colonization.

Due to geographical and historical circumstances, this archipelago in the middle of the North Atlantic became a pivotal region between Europe, Africa and America. This excellent location fostered the introduction of a wide variety of economically valuable plants, for acclimatization and further dissemination to other regions of the world. In fact, Cidade Velha (in Santiago), the former capital of the archipelago and the first town built in the tropics by Europeans (in the late 15th century), was an essential Atlantic port of call and rapidly became a commercial hub between Europe, Africa and the New World [49,50] promoting the introduction and later diffusion of many useful plants.

Overall, species were introduced to meet the needs of local populations, and the shifts over time reflect the changing in socio-economic requirements, from the most basic needs—food, fuelwood or timber—to higher-level demands, e.g., aesthetics.

### 3.3. Plant Uses and Sustainability

Ornamental followed by forage/pasture and food purposes are the primary uses of plants in Cabo Verde. The common use of ornamental plants in this archipelago is not surprising. The same occurs worldwide, with estimates pointing to 28,000 plant species of ornamentals (including gardening and landscaping plants), while cultivated crops correspond to about 7000 species [51]. However, it is worth mentioning that more than half of the species reported as ornamentals in Cabo Verde are also used for other purposes, namely for medicinal uses (data not included in the present analysis), food or for environmental projects.

Species used as forage or pasture, the second most reported use, are mainly from the families Poaceae and Fabaceae and are very common in grasslands and savannas. Several species are reported as high-quality forages (e.g., *Desmodium tortuosum* and *Teramnus labialis*), revealing their extraordinary value to improve natural pastures for cattle raising, a main economic sector in Cabo Verde [52].

Concerning edible species, it is interesting to note the presence of several commonly overlooked fruit-trees such as the introduced *Syzygium jambos* or *Spondias mombin*, and the reference to some native species whose fruits are used for human consumption such as *Momordica charantia*, *Grewia villosa*, *Ficus sur*, *F. sycomorus*, *Solanum scabrum* (leaves also used as vegetable) and the endemics *Phoenix atlantica* and *Sideroxylon marginatum*. The flour made from dry fruits of *Ziziphus mauritiana* is consumed by the populations living in dry areas, and, formerly, the fruits of *Tamarindus indica* were cooked together with meat [53]. Other native species used for food (vegetables) are *Launaea intybacea*, an ingredient of the most emblematic dish in Cabo Verde (“cachupa”), *Portulaca oleracea*, to make soups, *Celosia trigyna*, and the edible tubers of *Cyperus esculentus* and *C. rotundus*. *Senna occidentalis* was used, until recently, as a coffee substitute.

Particularly interesting are several native species reported as being used in times of food shortages (e.g., seeds used to make flour): the Malvaceae *Melhania ovata* and the Poaceae *Dactyloctenium aegyptium*, *Setaria barbata*, *Urochloa caboverdiana* and *U. ramosa*.

Note that several grass species occurring in Cabo Verde are considered as millets, a group of cereal crops with small grains used for human consumption. That is the case of the Guinea millet *Urochloa deflexa*, or the browntop millet *Urochloa ramosa*. Although abandoned many years ago, the millets are now being considered valuable functional foods for their good nutritional properties [7], and their use could be relevant to improve food security in arid regions owing to their ability to withstand adverse agroecological conditions [54].

Guinea millets, together with tubers, e.g., cassava, were the food base of island populations until the introduction of maize by the end of the 15th century/early 16th century [55]. These small grain crops are now uncommon, with maize (*Zea mays*) and beans (*Cajanus cajan*, *Lablab purpureus*, *Phaseolus lunatus*, *Ph. vulgaris* and *Vigna unguiculata*) being the prime food species in Cabo Verde [6].

Currently, maize and bean are the ingredients of traditional dishes: the “xerém” and “couscous”, prepared in different ways with maize, and “cachupa”, prepared with maize, several species of beans, cabbages, cassava and sweet potato.

Besides millets, other valuable small grain crops—*Amaranthus caudatus* and *A. cruentus*—are also present in Cabo Verde. These minor crops are presently underused but are becoming increasingly relevant as alternative crops in dry and semi-dry areas where major crops do not develop well [56], representing a promising resource to support food security.

In the early centuries of the archipelago’s colonization, several plants played crucial roles in the local economic activities. That was the case of native tanning plants, such as *Periploca chevalieri* and *Euphorbia tuckeyana* (tanned leather was one of the most reputed exports) and the orseille, *Roccella* spp. or *Ramalina* spp. (lichens, a taxonomic group not included in the present work), widely used to dye textiles (“panos da terra”) [55] and exploited in Cabo Verde since 1469 [57]. Other relevant productions were indigo (obtained from *Indigofera tinctoria*), extracted and used around the 16th century [58], cotton (*Gossypium* spp.), sugarcane (*Saccharum officinarum*) and the American physic nut (*Jatropha curcas*), whose seed oil was extracted to make soap or candles, all main exports during the 19th century [55]. Today, and except for sugarcane—used to produce “grogue” (an alcoholic spirit similar to rum), one of the main exports, produced in Santo Antão, São Nicolau and Santiago—and coffee (*Coffea* spp.)—in Fogo and São Nicolau islands (where it was introduced in 1778, [59]—these activities are almost completely abandoned.

Species used for utilitarian purposes include *Sida rhombifolia*, to make brooms; *Urena lobata* and *Calotropis procera*, to obtain fiber, with the latter used to fill mattresses and pillows (as reported by Roberts and Defoe [22]) as well as for firewood; *Phoenix atlantica* leaves, for basketry; *Cyperus alternifolius* subsp. *flabelliformis*, for mat weaving; and *Dichanthium annulatum* and *Imperata cylindrica*, for roofing.

For centuries, the continuous need for wood as a fuel for cooking and as a building material led to the overexploitation of the few native woody species and, therefore, to a severe reduction of their populations. There are interesting references in the early 18th century [22] to the over-exploitation of wild fig trees (probably *Ficus sycomorus* and *F. sur*) to build canoes, and of the dragon tree (*Dracaena caboverdeana*) to build houses in São Nicolau. Other widely used species were *Sideroxylon marginatum*, for timber, and *Arthrocaulon franzii*, *Calotropis procera*, *Dichrostachys cinerea*, *Echium vulcanorum*, *Launaea arborescens*, *Tamarix senegalensis* and *Tetraena gaetula* subsp. *waterlotii* for firewood.

It should be noted that the percentage of inhabitants currently using fuelwood is still quite significant, particularly in Fogo (50.1%), Santo Antão (39.6%), Santiago (30.9%) and Maio (30.5%) (see Supplementary Materials Table S2).

The social and cultural role of plants is also relevant in Cabo Verde, with particular species being of great symbolic value for ceremonial festivities. A few examples are the use of leaves of *Phoenix* spp. for religious ceremonies, such as at Easter time, or to decorate the streets to welcome governors in colonial times, or the use, in São Nicolau, of the endemic *Asteriscus smithii* in bonfires on Saint John’s eve.

To meet population needs, as well as to restore degraded land and improve soil characteristics, several woody species were introduced through afforestation programs, especially by the mid-20th century. In the highlands of Santo Antão, Santiago and Fogo, species of the genera *Eucalyptus*, *Hesperocyparis* and *Pinus*, among others, were and still are widely planted, while the lowlands are extensively afforested with the Southern American *Prosopis juliflora*, species of the genera *Acacia* and *Vachellia*, and *Ziziphus mauritiana*. To halt the erosion of slopes, several exotic species such as *Aloe vera*, *Lantana camara*, and *Furcraea foetida* were formerly used. Native grasses, such as *Heteropogon contortus* and *Bothriochloa bladhii*, are also reported as important species for this purpose, and their use may be a good alternative to consider.

With the increasing valuation of native species (e.g., Bozzano et al. [60]), the use of autochthonous resources in reforestation/afforestation programs is now being promoted by the Cabo Verdean authorities in charge of forestry—e.g., Direção Geral da Agricultura Silvicultura e Pecuária (DGASP-MAAP) and international agencies (such as the World Bank, the United States Agency for International Development, or the Global Climate Change Alliance). These species are better adapted to local conditions and more likely to enhance biodiversity and improve ecosystem services while providing traditional products (e.g., fruits, wood) to local communities.

Most of the useful species in Cabo Verde are introduced and/or distributed worldwide and, as such, they do not represent serious conservation issues, except for the environmental impacts resulting from the invasive behaviour of some of them. This is the case with *Lantana camara*, *Furcraea foetida*, *Prosopis juliflora* or *Eucalyptus* spp., which are currently seriously damaging native species and ecosystems.

More worrying is the overuse of some native species (e.g., the non-endemic *Tamarix senegalensis* or *Ficus* spp.) and, in particular, of the 38 endemic species listed in the present work, most of them endangered. Reported for all use types, they are relevant in the livelihood of Cabo Verdean population.

The end of some commercial activities (e.g., tanning, dyeing) and the switch from firewood to cooking gas, especially in rural households, have reduced anthropogenic pressure, but the sustainability of some native plant populations (e.g., *Sideroxylon marginatum, Dracaena caboverdeana*) is far from certain.

Finally, most ornamental species are introduced and do not raise conservation concerns. However, some native or even endemic species (e.g., *Echium* spp., *Nauplius* spp., *Phoenix atlantica* or *Aeonium gorgoneum*) are become increasingly attractive to local population as ornamentals and for other purposes, which may threaten their populations. This situation can be avoided, provided that cultivation is promoted and no pressure is imposed on wild populations. On this issue, it is interesting to note that schemes are being proposed (e.g., Krigas et al. [61]) to assess the potential of neglected or underused local endemic plants. Ensuring the safeguarding of native populations, which in Cabo Verde involves their prior recovery, will allow for the sustainable exploitation of these resources and benefit the local economy. In this respect, it is important to mention that several local initiatives, promoted by government agencies and non-governmental organizations (for example, the “Associação Projecto Vitó” on Fogo Island), have played a relevant role in informing and involving local populations in the safeguarding of threatened taxa.

## 4. Materials and Methods

### 4.1. The Study Area

Cabo Verde is a volcanic archipelago in the Atlantic Ocean, with 10 islands and several islets, about 600 km off the West African coast. The topography is generally very rugged, with high massifs and deep valleys. The island of Fogo reaches the highest elevation at 2829 m, followed by Santo Antão (1979 m), Santiago (1392 m), and São Nicolau (1304 m) [13].

A dry tropical climate with two well-marked seasons (humid and dry) and a limited and irregular rainfall (mean annual value about 300 mm) constrains the distribution of flora and vegetation. However, the topography contributes to significant spatial variations according to altitude and exposure to prevailing winds, leading to contrasting weather conditions [13,45]. Cabo Verde’s biodiversity is poor when compared to the other archipelagos of Macaronesia [62]. Native flora comprises about 400 taxa [63], of which 92 are endemic [32]. Plant communities are diversified and include open woodlands, scrubs, savannas and grasslands [33].

Among the main economic activities are livestock rearing (cattle, goats, poultry, pigs, rabbits, donkeys, and horses) and agriculture, with the latter limited to areas of adequate topographic and edaphoclimatic conditions [4,52].

To mitigate the effects of erosion, afforestation programmes were initiated in the 19th century and intensified during the 20th century, especially in mountainous areas of higher altitude islands (namely, Santo Antão, Santiago and Fogo) and in the more humid windward-facing slopes; the forestation of arid lowlands is more recent, dating from the second half of the 20th century [64,65].

### 4.2. Listing Useful Plants

A comprehensive review of the literature, including the Flora of Cabo Verde [66,67,68] and scientific publications (e.g., [11,15,16,25,29,31,63,69,70,71,72,73,74,75,76,77,78,79,80,81,82,83,84,85,86,87,88,89,90,91,92,93,94,95,96]), was undertaken to compile the list of useful plants in Cabo Verde and respective common names. We also examined several historical documents published between 1506 and the late 19th century, namely Valentim Fernandes 1506–1510 (in Monod et al. [18]), Gaspar Frutuoso, 1522–1591 (in Frutuoso [20]), Roberts and Defoe [22], Feijó (in Feijó [24], and Gardère et al. [25]), Forster [23], Chelmicki & Varnhagen [26,27], and Ficalho [58], which allowed us to identify the species used since the first settlements. These historical references help to understand how and when species were introduced and to provide a historical perspective on this subject. Information collected from herbarium specimens, especially LISC Herbarium (University of Lisbon), which hosts one of the most complete collections of Cabo Verdean plant species, was also used, as well as data collected in all the islands during field surveys performed by the authors (especially M.C.D., I.G., S.G., A.R., and M.M.R.) during the last decades.

Taxa nomenclature primarily follows Plants of the World Online [30]. Other databases, such as World Flora Online [34], were occasionally used. Note that there is still some uncertainty about the classification of some endemic taxa, which has led to frequent nomenclatural changes; the most relevant cases (for example, in the Apiaceae) are duly noted.

Information about growth form (habit) was obtained from taxa descriptions in the Flora of Cabo Verde and in World Flora Online [34]. Seven categories were considered: annual/biennial herbs, perennial herbs, shrubs/subshrubs (inc. rosette shrubs), trees (including palms and tree-like species), annual herbaceous climbers (annual vines), perennial herbaceous climbers (perennial vines) and woody climbers (lianas).

Eleven categories of uses (adapted from Cook [97]) were considered: food for humans (including beverages, food additives, spices, condiments, colorants, etc.); forage for livestock (including plants for pasture); materials (including plants producing gums, resins, oils, latex, waxes, tannins, dyes, etc.); timber; poison (e.g., for hunting and fishing, or used as biocides); melliferous; social uses (including stimulant, smoking materials, and plants used in ceremonial or ritual practices); fuelwood (firewood, charcoal); utilitarian (including plants used to make domestic utensils or tools, and sources of fibres); ornamental (garden plants, street trees, hedge plants, etc.); and environmental use (for revegetation, forestation and erosion control, as windbreaks, etc.). Medicinal species and gene source plants were not included in the present analysis.

Species distribution in Cabo Verde and worldwide was mainly based on the Flora of Cabo Verde [66,67,68], Sánchez-Pinto et al. [63] and Plants of the World Online [30]. Biogeographic distribution was established for each taxon using the regions established by Morrone [98] (Nearctic, Palaearctic, Neotropical, Afrotropical, Oriental, Australotropical, Andean, Afrotemperate, Antarctic, Neoguinean, Australotemperate and Neozelandic). When the species occurred in two or more regions, classification was based on the main distribution area(s). To avoid a large number of classes with a very low representation in Cabo Verde, some regions were merged (for details see the legend of Figure 6).

The conservation status was obtained from Romeiras et al. [37] and the IUCN Red List of Threatened Species [36].

### 4.3. Geographic and Socio-Economic Data

Fourteen indicators, summarized in Supplementary Materials Table S2, were selected to study the relationships between the diversity and distribution of useful flora in Cabo Verde with geographic, demographic, and economic factors. Data were obtained from official sources, produced by public authorities, namely Instituto Nacional de Estatística [14,99,100], Ministério da Agricultura e Ambiente [101] and Ministério do Desenvolvimento Rural [102].

### 4.4. Data Analysis

The relationships between the diversity and distribution of useful species and the geographical, demographic and economic indicators were analyzed using Spearman’s rank correlation coefficients. Correlation values were calculated using the “cor” function and the “Spearman” method. A probability value equal to or less than 0.05 was used to determine statistical significance. The visualization of the correlation matrix was produced with the packages “ggplot2” and “ggcorrplot” and the function “ggcorrplot”. All these statistical analyses were performed in R v. 4.0.5 [103].

The chord diagram showing the relation between the uses and the habit of the taxa was performed with the package “circlize” v.0.4.14 and the function “chordDiagram”.

The heatmap was produced based on the number of taxa with different use categories, for each island. This analysis was performed with the function “heatmap”, and the resulting chart was normalized by columns.

The treemap chart, providing a hierarchical view of the data, was created in Microsoft Excel v.2202.

## 5. Conclusions

This study shows that the biodiversity of Cabo Verde is threatened by human activities that meet the basic needs of local populations in the particularly harsh environment of this Macaronesian archipelago. This calls for the integration of scientific information with local indigenous knowledge, but comprehensive knowledge of the plants traditionally used is unavailable. Therefore, we used an integrative approach (i.e., (1) the taxonomic diversity and phytogeographic distribution of useful plants in Cabo Verde; (2) the role of geophysical, demographic, historical, economic, and socio-cultural factors on species distribution and uses; and (3) the potentially relevant species, particularly native ones) for the sustainable development of the archipelago. Information about the plant species and their uses was obtained for 11 categories of uses (e.g., food, forage/pasture, materials, timber, poison, melliferous, social, fuelwood, utilitarian, ornamental and environmental), and species biogeographic distributions and conservation status, as well as 14 geographic and socio-economic indicators, were also taken into account.

Cabo Verde was uninhabited before the mid-15th century, and many exotic plant species were introduced, first to ensure human survival and later also to support commercial interests. In this study, we identified 518 useful taxa (88 plant families), of which only 145 are native and 38 are endemic. Taxa of Afrotropical origin prevail as forage, whereas ornamentals are mainly Neotropical. Only less than 38% of the recorded taxa were assessed by the IUCN Red List, and they include 44 species classified with some level of threat. The number of useful taxa is correlated with altitude and agricultural area, as well as with the size of the rural population, number and type of farms, but not with total population or socio-economic indicators such as the number of tourists or gross domestic product. Plants are primarily used as ornamentals and for forage and food; native taxa—including many threatened ones—are mostly used for fuelwood, forage and utilitarian purposes. The islands where agricultural activities prevail present more useful taxa than the others. The agrobiodiversity and traditional practices (e.g., multiple cropping, selection of resistant varieties and storage of propagules) are crucial to cope with recurrent droughts and to ensure food security in Cabo Verde. Our inventory discloses previously ignored information on particular species; in particular, some species are no longer used.

This study improves the knowledge of the useful plants of Cabo Verde. Most of the useful species are introduced but do not represent serious conservation problems; of much more concern is the overuse of some native taxa and, in particular, of 38 endemics listed, most of them endangered. Only by ensuring the safeguard of native plant populations in Cabo Verde will the sustainable exploitation of these resources be possible and benefit the local economy.

## Figures and Tables

**Table 1:** Native useful taxa occurring in Cabo Verde, including information on family, common names (mostly in creole), main uses (Fd, food; Fr, forage; Ml, melliferous; P, poison; S, social uses; Fu, fuelwood; T, timber; U, utilitarian uses; Mt, materials; O, ornamental; E, environmental use; for details, see Materials and Methods), and historical use (Hist.). Taxonomic authorities according to Plants of the World Online [30].

Taxa	Common Names	Uses	Hist.
**Acanthaceae**			
*Dicliptera verticillata*	Joelho, orelha-de-rato, rapazinho	Fr	
**Aizoaceae**			
*Zaleya pentandra*	Erva-de-rapé	S,O	
**Amaranthaceae**			
*Aerva javanica*	Florinha, panasco	U	
*Amaranthus graecizans* subsp. *graecizans*	Bredo, bredo-femba	Fr	
*Arthrocaulon franzii*	Murraça, murraça-rosa-crioula, murraçona	Fu	
*Celosia trigyna*		Fd	
*Patellifolia procumbens*	Selga	Fr	
**Apiaceae**			
*Tornabenea annua [a]	Funcho, funtcho, futcho-bravo	Fr	
*Tornabenea tenuissima [a]	Aipo, funtcho	Fr	
*Tornabenea bischoffii [a]	Funcho	Fr	
**Apocynaceae**			
*Calotropis procera*	Bombardeiro	Fr,Fu,U	●
*Periploca chevalieri	Corcabra, curcabra, lantisco, lentisco	Fr,Mt	●
**Arecaceae**			
*Phoenix atlantica	Tamareira	Fd,Fr,U,O,E	
**Asparagaceae**			
*Dracaena caboverdeana [b]	Dragoeiro	Mt,O,E	●
**Asteraceae**			
*Artemisia gorgonum	Losna	P	
*Asteriscus daltonii subsp. *vogelii*	Macela, marcela, marcela-lenha	P	
*Blainvillea gayana*	Barba-bodi, cachacinho, erva-moura, loura, targa, targa-branco	Fr	
*Conyza feae [c]	Losna-brava, marcela, marcelinha, mato-contrário, palha-santa	Fr	
*Launaea arborescens*	Carqueja, craquejo	Fu	
*Launaea intybacea*	Algodão-de-garça, paja-leite, palha-de-leite, serralha	Fd	
*Launaea picridioides	Marê-tope, serragem, serralha, tortolhinha, tortolhinho	Fr	
*Launaea thalassica	Serralha, tortolhinha	Fr	
*Pseudoconyza viscosa*	Butra, talga, vampiro	Fr	
*Pulicaria diffusa	Losna	P	
*Sonchus daltonii	Coroa-de-rei	Fr	
*Sonchus oleraceus*	Algodão-de-graça, leituga, palha-leite, sarralha, serralha	Fr	
*Vernonia colorata*	Catchiça	Fr	
**Boraginaceae**			
*Echium hypertropicum	Língua-de-vaca, língua-di-baca	Fr,Fu	
*Echium stenosiphon subsp. *glabrescens*	Língua-de-vaca	Fr	
*Echium stenosiphon subsp. *lindbergii*	Língua-de-vaca	Fr	
*Echium stenosiphon subsp. *stenosiphon*	Língua-de-vaca	Fr	
*Echium vulcanorum	Língua-de-vaca	Fr,Fu	
*Heliotropium ramosissimum*	Alfavaca, alfavaca-da-achada, erva-das-sete-sangrias, furtaragem, mama-de-bitcho, tchero-fede, três-marias	Fr	
**Brassicaceae**			
*Diplotaxis glauca	Matona, mostarda, mostarda-braba	Fr	
*Diplotaxis varia	Mostarda-braba	Ml	
*Lobularia canariensis subsp. *spathulata*		O	
*Lobularia canariensis subsp. *fruticosa*	Sempre-noivinha	Ml,O	
**Caryophyllaceae**			
*Polycarpaea gayi	Cidreira-de-rocha, mato-branco, palha-bidião, palha-de-bidion, talim, talinho, telim	U	
**Cistaceae**			
*Helianthemum gorgoneum	Matinho, piorno-de-flor-amarela	Fr	
**Commelinaceae**			
*Commelina benghalensis*	Grande-rato, orelha-de-rato, palha-de-água	Fr	
**Convolvulaceae**			
*Distimake aegyptius*	Maraganha, n’onhen’onhe, palha-corda	Fr	
*Ipomoea eriocarpa*	Cordinha, jejé-calabaceira, lagaço-cozinho, legação-cabecinho, monhe-monhe, monho-monho	Fr	
*Ipomoea pes-caprae* subsp. *brasiliensis*	Lacacã, lacacã-grande, lacacan-de-vaca, legação-de-rocha	E	
**Crassulaceae**			
*Aeonium gorgoneum	Ceilão, mata-sede, saião, seilão, sião	O	
**Cucurbitaceae**			
*Citrullus colocynthis*	Balancia-brabo, melancia-brava, melão-bravo, olho-de-boi, olho-de-vaca	Fr	
*Momordica charantia*	Aboboreira-de-são-caetano, banana-rato, erva-de-são-caetano, palha-de-são-caetano, sancaetano, são caetano	Fd,Ml,Mt	
**Cyperaceae**			
*Bulbostylis barbata*	Soldinha	Fr,Ml	
*Cyperus alternifolius* subsp. *flabelliformis*	Chapeudisol, goia, junco	Fr,U	
*Cyperus articulatus*	Goia, junco	Fr,U	
*Cyperus esculentus*	Djunça, junça, vista	Fd,Fr	
*Cyperus hortensis*		Fr,U	
*Cyperus rotundus*	Grama, guel, injunça, junça, junco	Fd,Fr	
*Fimbristylis ferruginea*	Junco, junquinho	Fr	
**Equisetaceae**			
*Equisetum ramosissimum*	Carsim, cavalinha, talim	S	
**Euphorbiaceae**			
*Euphorbia tuckeyana	Tira-olho, tortilho, tortodjo, tortolho	Fu,Mt	●
**Fabaceae**			
*Abrus precatorius* subsp. *africanus*	Jequeriti, santa-clara	Fr,U	
*Acacia caboverdeana [b]	Espinheiro-branco, neu-neu (fruits)	Fr,Fu,E	●
*Alysicarpus ovalifolius*		Fr	
*Clitoria ternatea*	Bachinha-de-cordoniz, palha-lopes	Fr	
*Crotalaria senegalensis*	Ovos-de-rato, ovos-de-rato-pequeno	Fr	
*Desmodium ospriostreblum*	Crioulinha	Fr	
*Dichrostachys cinerea*	Espinheiro, espinheiro-branco, espinheiro-cachupa, espigo-de-cachupa, espinho-cachupa, espinho-catchupa	Fu	
*Genista stenopetala*		E	
*Grona hirta*	Maratchinga, marquinha	Fr,E	
*Lablab purpureus* subsp. *purpureus*	Creca, feijão-branco-de-vagem-branca, feijão-caqui, feijão-careca, feijão-cutelinho, feijão-pedra, feijão-pedra-bombone, feijão-vaca	Fd,Fr	●
*Lotus brunneri	Cabritagem, cafetalha, cafetagem, piorno-amarelo, piorno-preto	Fr,P	
*Lotus jacobaeus	Piorno, piorno-preto	Fr	
*Lotus purpureus	Piorno, piorno-amarelo	Fr	
*Macrotyloma daltonii*	Corda-lopes, cordeirinha-preta, favalinha, feijoeiro-de-lagartiga	Fr	
*Rhynchosia minima* var. *memnonia*	Feijoeiro-de-lagartixa	Fr	
*Sesbania pachycarpa*	Acácia-sizinanthe, sesinanthe, ticome-se	Fr	
*Stylosanthes fruticosa*		Fr	
*Tephrosia linearis*		Fr	
*Tephrosia purpurea*		Mt	
*Teramnus labialis* subsp. *arabicus*	Caransaqui, corda-lopes-pequena, cordeirinha-branca	Fr	
*Vachellia nilotica* subsp. *adstringens*	Acácia	Ml,O,E	
*Vigna unguiculata* subsp. *unguiculata*	Bongolon-d’angola, feijão-bezugo, feijão-bongolon, feijão-bongolon-amarelo, feijão-bongolon-com-boca-preta, feijão-congo	Fd,Fr	●
**Frankeniaceae**			
*Frankenia caboverdeana [b]	Mato-de-engodo, mato-de-sargaço, palha-engodo	U	
**Lamiaceae**			
*Lavandula coronopifolia*	Marmulano-da-terra, risco, risque	Fr	
*Lavandula rotundifolia	Aipo, alfazema-brava, gilbon	Fr	
*Ocimum americanum*		Fd	
*Salvia aegyptiaca*	Alfazema, alfazema-da-terra,bálsamo-de-pastor, ermofassima, malfazema, marcelina, rosmaninho	Fr	
**Malvaceae**			
*Grewia villosa*	Balneda, barnadeiro, barnedo, barneldo, barnelo	Fd,Ml,U	
*Melhania ovata*	Lolo-branco, mato-branco, salva-vidas	Fd	
*Sida rhombifolia*	Lolo, loulo, loulo-preto-grande	Fr,U	
*Urena lobata*	Bassago	U	
**Moraceae**			
*Ficus sur*	Figueira, figueira-brava, figueira-preta	Fd,Fr,T	●
*Ficus sycomorus*	Figueira-branca, figueira-brava, figueira-de-figos-grandes	Fd,Fr,T,E	●
**Nyctaginaceae**			
*Boerhavia coccinea*	Albeza, batata-de-asno, batata-de-burro, batata-de-oze, cordeira, costa-branca, costa-branca-fêmea, mato-branco	Fr	
*Boerhavia diffusa*	Albeza, batata-de-burro, costa-branca, costa-branca-fêmea	Fr,Ml	
*Boerhavia repens*	Costa-branca, costa-branca-fina, costa-branca-miúda, folha-branca, palha-branca, palha-costa, palha-seca	Fr	
*Commicarpus helenae*	Albeça-branca, albéza-branco, butra, costa-branca, costa-branca-bastarda, folha-branca, mato-branco, mato-lagarto	Fr	
**Plantaginaceae**			
*Globularia amygdalifolia	Argueiro, mato-botão, medronho	Fr	
**Poaceae**			
*Andropogon gayanus* var. *tridentatus*	Palha-ladeira, touça, touça-fêmea	Fr	
*Bothriochloa bladhii*	Touça, touça-macho, palha-cavalo	Fr,E	
*Cenchrus ciliaris*	Balanco, palha-branca, palha-grossa, rabo-de-gato	Fr	
*Cenchrus pedicellatus* subsp. *pedicellatus*	Balanco-branco	Fr	
*Cenchrus pedicellatus* subsp. *unispiculus*	Balanco-branco	Fr	
*Chloris gayana*		Fr	
*Chloris pilosa*		Fr	
*Dactyloctenium aegyptium*	Djinguilano, jéjé-jiuguilam, palha-de-boi-fraca, pé-de-galinha	Fd,Fr	
*Dichanthium annulatum*	Palha-soca, soca, touça-fêmea	Fr,U	
*Dichanthium foveolatum*	Palha-fina, palha-minha, palha-sisuda, sisuda	Fr	
*Digitaria ciliaris*	Djé-djé-cinha, djé-djé-pequeno, djeiezinho	Fr	
*Digitaria horizontalis*	Gé-gé, jéjézinho	Fr	
*Digitaria nodosa*	Palha-carriço, palha-grossa	Fr	
*Echinochloa colonum*	Djé-djé-pequeno	Fr	
*Eleusine indica* subsp. *indica*	Barba-de-bode, palha-boi, palha-grossa	Fr	
*Eragrostis cilianensis*	Djé-djézinho	Fr	
*Eragrostis ciliaris*	Padja-do-menino-jesus, palhinha	Fr,U	
*Eragrostis minor*		Fr	
*Hackelochloa granularis*		Fr	
*Heteropogon contortus*	Azagaia, rabo-de-asno, soca-mansa, touça-mansa, toussa-matcho	Fr,E	
*Heteropogon melanocarpus*	Zagaia	Fr	
*Hyparrhenia caboverdeana [b]	Palha-de-guiné, palha-negra, touça, touça-fêmea	Fr	
*Imperata cylindrica*	Palha-carga	Fr,U	●
*Melinis minutiflora*	Palha-governo, palha-mafe, palha-mafó	Fr	
*Paspalum scrobiculatum*	Patacho	Fr	
*Polypogon viridis*	Graminho, palha-de-água	Fr	
*Rottboellia cochinchinensis*	Palha-grossa	Fr	
*Schizachyrium brevifolium*		Fr	
*Setaria barbata*	Djé-djé-palha-de-água, jéjé	Fd,Fr	
*Setaria pumila*	Gé-gé-pequeno	Fr	
*Setaria verticillata*	Pega-saia	Fr	
*Tricholaena teneriffae*	Palha-branca, palha-de-vassoura	Fr	
*Urochloa caboverdiana	Dje-dje, jé-jé	Fd,Fr	
*Urochloa ramosa*	Djé-djé, jé-jé, jé-jé-folha-larga	Fd,Fr	
*Urochloa xantholeuca*	Djé-djé	Fr	
**Portulacaceae**			
*Portulaca oleracea*	Beldroega, bordulega, brêdo-fêmea, sangue-sangria	Fd,Fr,Ml	
**Pteridaceae**			
*Adiantum capillus-veneris*	Aibenca, avenca	O	
**Resedaceae**			
*Caylusea hexagyna*	Campa, laca-laca, laga-laga, palha-lagada, piorno	Fr,Ml	
**Rhamnaceae**			
*Ziziphus mauritiana*	Simbrom, zimbrão, zimbreiro-da-índia	Fd,Fr,Ml,Fu,T,E	
**Rubiaceae**			
*Oldenlandia corymbosa* var. *corymbosa*		Fr	
**Sapindaceae**			
*Cardiospermum halicacabum*	Conta-de-cavalo	O	
*Dodonaea viscosa*		O	
**Sapotaceae**			
*Sideroxylon marginatum	Figueira-de-macaco, marmulano, marmolano	Fd,Fr,Fu	●
**Solanaceae**			
*Solanum nigrum*	Malagueta-de-galinha, uva-catchorro, uva-de-santa-maria	Fd	
*Solanum scabrum*		Fd	
**Tamaricaceae**			
*Tamarix senegalensis*	Tarafe, tarrafe, tamargueira	Fu,O,E	●
**Typhaceae**			
*Typha domingensis*	Palha-das-esteiras, tabúa	U	
**Urticaceae**			
*Forsskaolea procridifolia	Língua-de-vaca-branca, mato-gonçalo, ortiga, palha-renda, pega-saia, rafa-saia, rapa-saia, urtiga	Fr	
**Zygophyllaceae**			
*Fagonia cretica*	Arroz-de-pardal, cabritaia-do-campo, matinho-de-agulhas	Fr,P	
*Fagonia latifolia* [c]	Cabaceira, matinho	Fr	
*Tetraena gaetula* subsp. *waterlotii* [c]	Acelga-de-água, bidion, fuminga, morraça-branca, murraça	P,Fu,E	
*Tetraena vicentina		E	

[a] In the absence of a comprehensive review of all the endemic Apiaceae occurring in Cabo Verde, we follow Brochmann et al. [31] and Romeiras et al. [32]. [b] According to Rivas-Martínez et al. [33]. [c] According to World Flora Online [34]. * Endemic taxa. ● Taxa with reported historical use.

**Table 2:** Introduced useful taxa occurring in Cabo Verde, including information on family, common names (mostly in creole), main uses (Fd, food; Fr, forage; Ml, melliferous; P, poison; S, social uses; Fu, fuelwood; T, timber; U, utilitarian uses; Mt, materials; O, ornamental; E, environmental use; for details, see Materials and Methods), and historical use (Hist.). Taxonomic authorities according to Plants of the World Online [30].

Taxa	Common Names	Uses	Hist.
**Acanthaceae**			
*Eranthemum pulchellum*	Flor-viúva	O	
*Pseuderanthemum maculatum*	Dakarense	Ml	
**Aizoaceae**			
*Carpobrotus edulis*	Bálsamo	O,E	
*Tetragonia tetragonioides*	Espinafre-da-nova-zelândia	Fd	
**Amaranthaceae**			
*Alternanthera sessilis*	Abri-olho, abrodjo, arre-porra, mão-na-pé, mon-na-pé	Fr,O	
*Amaranthus blitum*	Bredo	Fd	
*Amaranthus caudatus*	Bredo-macho	Fd,Fr,O	
*Amaranthus cruentus*	Crista-de-perú	Fd,Fr	
*Amaranthus hybridus* subsp. *hybridus*	Bredo-macho, rabo-de-galo	Fr	
*Amaranthus spinosus*	Bredo, bredo-com-espinhos, bredo-espinhoso, bredo-macho	Fd	
*Amaranthus tortuosus*	Bredo, bredo-macho	Fr	
*Amaranthus viridis*	Bredo-sem-espinhos	Fd,Fr	
*Atriplex halimus*		Fr,O,E	
*Beta vulgaris*	Beterraba	Fd,Fr,Mt	
*Gomphrena globosa*		O	
**Amaryllidaceae**			
*Allium ampeloprasum*	Alho-francês	Fd	
*Allium ascalonicum*	Chalota	Fd	
*Allium cepa*	Cebola	Fd	
*Allium fistulosum*	Cebolinha	Fd	
*Allium sativum*	Alho	Fd	
*Allium schoenoprasum*	Cebolinha-miúda	Fd	
*Hymenocallis littoralis*	Lírio	Ml,O	
*Scadoxus multiflorus*		O	
**Anacardiaceae**			
*Anacardium occidentale*	Cadju, cajueiro, cajuleiro	Fd,Ml,T,E	
*Mangifera indica*	Mangue, mangueira	Fd,Fr,Ml	
*Schinus molle*	Pimenteira, pimenteira-bastarda, pimenta-rosa	Fd,P,O,E	
*Schinus terebinthifolia*	Pimenteira	O	
*Sclerocarya birrea* subsp. *caffra*	Ocanho	Fd	
*Spondias mombin*	Mamipreiro, manipo	Fd,Ml	●
**Annonaceae**			
*Annona cherimola*	Cherimolia	Fd	
*Annona muricata*	Pinha, pinhão, pinhão-azedo, sap-sap	Fd,O	
*Annona reticulata*	Anoneira, coração-de-boi	Fd,O	
*Annona squamosa*	Pinha, pinho	Fd	●
**Apiaceae**			
*Anethum graveolens*	Endro, ente, entro, erva-doce	Fd,Fr	
*Apium graveolens*	Aipo	Fd	
*Coriandrum sativum*	Coentro, cuentro	Fd	
*Daucus carota*	Cenoura	Fd	
*Foeniculum vulgare*	Erva-doce, funcho, funcho-gomado	Fd	●
*Petroselinum crispum*	Salsa	Fd	
**Apocynaceae**			
*Asclepias curassavica*	Cravo, pitchula-de-leite	Ml,O	
*Cascabela thevetia*	Chapéu-de-napoleão, mundium	Ml,O	
*Catharanthus roseus*	Bigalo, flor-de-anjo, flor-de-finado, sempre-noiva	Ml,O	
*Nerium oleander*	Cevadilha, loendro, loureiro-rosa, rosa, rosa-loira, roseira-branca-singela, sempre-noiva-branca, sevadilha	P,O	
*Plumeria rubra*		O	
**Araceae**			
*Caladium bicolor*		O	
*Colocasia esculenta*	Inhame, mafafa, malanca, muncoco	Fd	
*Xanthosoma sagittifolium*	Inhame, mafafa-preta, malanca, mincoco	Fd	
**Arecaceae**			
*Borassus flabellifer*	Cibe	O	
*Cocos nucifera*	Coqueiro	Fd,Ml,O,E	●
*Elaeis guineensis*	Coconote, dem-dem, palmeira-do-azeite	Ml,O	
*Phoenix canariensis*		O	
*Phoenix dactylifera*	Palmeira-do-saará, tamareira, tamareira-do-saará	Fd,O,E	●
*Washingtonia filifera*	Palmeira-leque	O	
**Aristolochiaceae**			
*Aristolochia littoralis*		O	
**Asparagaceae**			
*Agave americana*		Ml	
*Agave sisalana*	Carapate-manila, carrapato-de-lisboa, pita, sisal	P,U,O	
*Asparagus officinalis*	Espargo	Ml	
*Dracaena hyacinthoides*		O	
*Furcraea foetida*	Carapate, carrapato, carrapato-da-terra, piteira-de-cabo-verde	P,U,O,E	●
**Asphodelaceae**			
*Aloe vera*	Aloés, babosa	Ml,S,O,E	
**Asteraceae**			
*Bidens bipinnata*	Gúia, seta, seta-branca, seta-preta	Ml	
*Bidens pilosa*	Agulha, gua, palha-agulha, seta, seta-preta, setinha	Fr,Ml	
*Calendula arvensis*		O	
*Cichorium endivia*	Endivia	Fd	
*Cichorium intybus*	Chicória	S,O	
*Helianthus annuus*	Girassol	Ml	
*Lactuca sativa*	Alface	Fd	
*Synedrella nodiflora*	Targa	Fr,Ml	
*Tagetes erecta*	Cravo, cravo-branco, cravo-de-burro	Ml,O	
*Tanacetum parthenium*	Altamires	O	
*Urospermum picroides*	Palha-leite, palha-leite-amarga, raposade, serralha	Fr,Ml	
*Zinnia peruviana*	Cravo, cravo-branco, zinha, zinia	Fr,O	
**Basellaceae**			
*Anredera cordifolia*		O	
*Basella alba*	Tinta-de-macaca, tinta-de-macaco	Mt,O	
**Bignoniaceae**			
*Crescentia cujete*	Cabaceira, calabaceira	U	
*Dolichandra unguis-cati*	Unha-de-gato	O	
*Handroanthus impetiginosus*	Pau-d’arco	S,Fu,Mt,O	
*Jacaranda mimosifolia*	Jacandrão	O,E	
*Kigelia africana* subsp. *africana*		O	
*Spathodea campanulata*	Árvore-da-chama, tulipeira-do-gabão, tulipa-do-gabão	O	
*Tabebuia rosea*	Farroba	O,E	
*Tecoma stans*	Ervilha-de-flor	O	
**Boraginaceae**			
*Cordia sebestena*		O	
*Heliotropium arborescens*	Baunilha, baunilha-de-cheiro	O	
**Brassicaceae**			
*Barbarea verna*	Agrião-de-terra	Fd	
*Brassica juncea*	Mostarda	Fd	
*Brassica nigra*	Mostarda, mostarda-branca, mostarda-brava, mostarda-preta	Fd,Ml	
*Brassica oleracea*	Couve	Fd,Fr	
*Brassica rapa*	Couve-chinesa, nabo	Fd	
*Eruca vesicaria*	Rúcula	Fd	
*Lobularia maritima*	Sempre-noiva	O	
*Matthiola maderensis*		O	
*Nasturtium officinale*	Agrião, agrião-de-água, agrião-vulgar	Fd	
*Raphanus raphanistrum* subsp. *sativus*	Rábano, rabanete	Fd	
**Bromeliaceae**			
*Ananas comosus*	Ananaseiro	Fd	●
**Cactaceae**			
*Opuntia ficus-indica*	Figueira-da-índia, figueira-do-inferno, tabaibo	Fd,Fr,Ml,O	●
*Pereskia aculeata*		O	
*Selenicereus undatus*	Barse, pilahayo	Fd,O	
**Calophyllaceae**			
*Mammea americana*	Abricó-do-pará, mamão, mamoeiro	Fd	●
**Cannaceae**			
*Canna indica*	Cana-da-índia, coqueirinho, coqueirinho-de-jardim, lírio	O	
**Caprifoliaceae**			
*Lonicera confusa*	Madressilva, madressilva-de-cheiro	O	
**Caricaceae**			
*Carica papaya*	Bijagó-preta, papaeira	Fd	●
**Caryophyllaceae**			
*Dianthus caryophyllus*		O	
**Casuarinaceae**			
*Allocasuarina verticillata*		E	
*Casuarina equisetifolia*	Casuarina	E	
**Combretaceae**			
*Terminalia catappa*	Amendoeira, amendoeira-da-índia	Fd,T,O	
**Commelinaceae**			
*Tradescantia zebrina*		O	
**Convolvulaceae**			
*Argyreia nervosa*		O	
*Ipomoea batatas*	Batata, batata-belém, batata-doce, batata-doce-preta, batata-malevinho, batata-quarenta-dias, corda-copo, cordinha, giginha-muralha, nhá-júlia, pau-de-vinho, quirino, temerosa	Fd,Fr,Ml	●
*Ipomoea carnea*		O	
*Ipomoea muricata*	Calabaceira	O	
*Ipomoea tuberculata*	Rosas-de-madeira	Fr,O	
**Crassulaceae**			
*Kalanchoe daigremontiana*	Bálsamo	O	
*Kalanchoe pinnata*	Bálsamo, figueirinha	O	
**Cucurbitaceae**			
*Citrullus lanatus*	Melancia	Fd	●
*Cucumis anguria*	Pepino-bravo, pepino-de-macaco, pepino-sanjo, pepino-santcho	Fr	
*Cucumis melo*	Melão	Fd	●
*Cucumis sativus*	Pepino	Fd	
*Cucurbita maxima*	Abóbora-roca, aboboreira, roca	Fd,Ml	
*Cucurbita moschata*	Abóbora-de-sequeiro-de-porco, abóbora-jarda, aboboreira	Fd,Ml	
*Cucurbita pepo*	Aboboreira, aboboreira-vulgar	Fd,Ml	●
*Lagenaria siceraria*	Abobreira-de-cabaça, buli, cabaça, cabaceira	Fd,U	
*Luffa aegyptiaca*	Bobra	U	
**Cupressaceae**			
*Cupressus sempervirens*	Cupressus	Fu,E	
*Hesperocyparis arizonica*		E	
*Hesperocyparis lusitanica*	Cedro-português, cedro-do-buçaco	Fu,E	
*Hesperocyparis macrocarpa*		E	
**Dioscoreaceae**			
*Dioscorea japonica*		Fd	
**Euphorbiaceae**			
*Acalypha wilkesiana*		O	
*Breynia disticha*	Groselhinha	O	
*Euphorbia chamaesyce*	Solda-inglesa	Fr,P	
*Euphorbia heterophylla*	Travador	Ml	
*Euphorbia hirta*	Desfamador, erva-santa-luzia, itervina, marcelinha, marcelintra, palha-pico, solda-inglesa, solda-inglesa-grande	Fr,Ml	
*Euphorbia hyssopifolia*	Padja-lete, palha-leite, palha-soda, solda-inglesa	Fr	
*Euphorbia milii*	Coroa-de-cristo	Ml	
*Euphorbia pulcherrima*		O	
*Euphorbia splendens*		O	
*Euphorbia tirucalli*	Borracha, mato-leitoso	O	
*Euphorbia tithymaloides*		O	
*Jatropha curcas*	Purga, purgueira	Mt,E	●
*Jatropha gossypiifolia*	Chagas-velhas, purgueira, purgueira-da-guiné	Fr	
*Jatropha multifida*	Purgueira-da-guiné	O	
*Manihot carthagenensis* subsp. *glaziovii*	Borracheira, mandioqueira-borracha	Mt,O	
*Manihot esculenta*	Mandioca, mandioca-borracha, mandioca-branca, mandioca-brasil, mandioca-mulata, mandioca-pau-de-terra	Fd,Fr,Ml	●
*Ricinus communis*	Bafureira, djague, djague-djague, jag-jag, mamona, rícino	Fr,Ml	●
**Fabaceae**			
*Acacia bivenosa*		E	
*Acacia brachystachya*		E	
*Acacia cyclops*		E	
*Acacia holosericea*	Alosericia, oredjona	Ml,E	
*Acacia longifolia*		E	
*Acacia mearnsii*		E	
*Acacia pycnantha*		E	
*Acacia salicina*		E	
*Acacia saligna*		E	
*Acacia victoriae*		E	
*Adenanthera pavonina*	Coral	O	
*Albizia lebbeck*	Pau-feijão	Ml,O,E	
*Arachis hypogaea*	Amendoim, mancarra	Fd,Fr	●
*Bauhinia galpinii*		O	
*Bauhinia monandra*		O	
*Caesalpinia pulcherrima*	Barbas-de-barata, brinco-de-princesa	O	●
*Cajanus cajan*	Congo, feijão-congo, feijão-ervilha, feijão-figueira	Fd,Fr	●
*Canavalia ensiformis*	Fava-rica	Fd,Fr	
*Cassia fistula*	Canafístula, canafrista, jardim	O	●
*Ceratonia siliqua*	Alfarrobeira	Fd,Fr,E	●
*Chamaecytisus prolifer*		Fr	
*Colophospermum mopane*		O	
*Crotalaria retusa* var. *retusa*	Bons-dias, flor-de-lagartixa, gaivé, ovos-de-rato	Fr	
*Delonix regia*	Acácia-rubra	Ml,O	
*Desmanthus virgatus*	Bencaiumba, bom-de-caimbra, caiumbra, quintinha, sementinha	Fr,Ml	
*Desmodium tortuosum*	Crioula, crioula-fina, crioula-pequena, marquinha	Fr	
*Erythrina variegata*		O	●
*Erythrina velutina*	Fabatera	O	
*Erythrostemon gilliesii*	Barbas-de-barata	Ml,O	
*Gliricidia sepium*		Fr,O	
*Guilandina bonduc*	Ouri, uri, uriseira	U	
*Indigofera tinctoria*	Tinta	Mt	●
*Leucaena leucocephala*	Acácia, acácia-leucena, linhaça, linhacho, sementinha-da-terra	Fr,Ml	
*Libidibia coriaria*	Crisalpina	Mt,O	
*Lonchocarpus sericeus*		O	
*Medicago sativa*	Anafe, luzerna	Fr	
*Millettia thonningii*		O	
*Mucuna pruriens*	Canhoma, feijão-de-bitcho, feijão-de-lagartixa, ganhoma	Fr	
*Parkia biglobosa*	Alfarroba-da-guiné	O	
*Parkinsonia aculeata*	Acácia, acácia-espinheiro, acácia-martins, espinho-branco	Fr,Ml,E	
*Phaseolus lunatus*	Banjona, bonjinho, fava, fava-terra, favona, feijão, feijão-bombone-branco, feijão-bonge, feijão-espadinha, feijão-fava	Fd	●
*Phaseolus vulgaris*	Bonje, favona, feijão, sapatinha	Fd	●
*Pithecellobium dulce*	Mampisa, roseira	Fd,O	
*Prosopis chilensis*		E	
*Prosopis juliflora*	Acacia-americana, algaroba	Fr,Ml,O,E	
*Prosopis pallida*		E	
*Prosopis tamarugo*		E	
*Samanea saman*	Pau-feijão	Fd,Fr	
*Senegalia senegal*		E	
*Senna artemisioides* nothosubsp. *sturtii*		E	
*Senna bicapsularis*	Beijinho, canafístula, flor, jardim, jardineira	O	
*Senna corymbosa*		O	
*Senna obtusifolia*		O	
*Senna occidentalis*	Baguinha, canafista, empincheira, fedegosa, munhanóca, pincheira, trincheira	Ml,S	
*Senna septemtrionalis*		O	
*Senna siamea*		O	
*Sesbania grandiflora*	Cacia-japónica	Fd,Ml,O	
*Tamarindus indica*	Tamarindeiro, tamarindo, tambarindo, tambarino	Fd,Fr,Fu,O,E	●
*Tara spinosa*	Tara-do-chile	O	
*Tipuana tipu*		O	
*Trifolium glomeratum*	Bonança, trevo	Fr	
*Vachellia farnesiana*	Acácia-esponja, aroma, espinheiro-branco, espinheiro-preto, espinho-branco, espinho-preto, esponjeira, imbulda, perfume	Ml,Mt,O,E	●
*Vachellia nilotica* subsp. *indica*	Acácia, espinheira, espinheiro-preto, espinho-preto	Fr,Fu,Mt,O,E	
*Vachellia seyal*		E	
*Vachellia tortilis*		E	
**Geraniaceae**			
*Pelargonium* × *hybridum*		O	
*Pelargonium inquinans*		O	
*Pelargonium zonale*	Malva-sardinheira	O	
**Iridaceae**			
*Iris florentina*	Lírio-branco, tulipa-branca	O	
**Lamiaceae**			
*Clerodendrum speciosissimum*	Rosaquina, rosa-quina	O	
*Clerodendrum umbellatum*		O	
*Lavandula dentata*	Rosmaninho	O	
*Leonurus sibiricus*		O	
*Mentha* × *wirtgeniana*	Bergamota, hortolô, hortelã	Fd	
*Mentha pulegium*	Poeijos	Fd	
*Mentha* x *piperita*	Ortelã-pimenta	Fd	
*Ocimum basilicum*	Mangericão, mangerona, mangirão, mangirona	Fd	●
*Ocimum gratissimum*		Fd	
*Origanum vulgare*	Mangerona-selvagem	Fd	
*Salvia coccinea*	Trepadeira-de-lisboa	O	
*Salvia eriocalyx*	Salva	O	
*Salvia leucantha*		O	
*Salvia rosmarinus*	Alecrim, alecrim-de-portugal	Fd	
*Tectona grandis*	Teca	T	
*Volkameria aculeata*		O	
**Lauraceae**			
*Cinnamomum burmanni*	Caneleira	Fd	
*Cinnamomum camphora*	Árvore-de-cânfora, canforeira	O	
*Cinnamomum verum*	Caneleira	Fd,O	
*Laurus nobilis*	Loureiro	Fd,P	
*Persea americana*	Abacate, abacateiro	Fd,Ml	
**Loasaceae**			
*Mentzelia aspera*	Lapadeira, pega-saia, rapo-tchapo, rato-tchapo	Ml	
**Lythraceae**			
*Punica granatum*	Romã, romangeira, romanzeira, romãzeira	Fd,Mt,O	●
**Malvaceae**			
*Abelmoschus esculentus*	Quiabo	Fd	●
*Abutilon grandifolium*	Vara-de-lobo	Mt	
*Adansonia digitata*	Calabaceira, caxabuceira, imbondeiro	Fd,Ml	●
*Ceiba pentandra*	Poilão, polon	Fd,Ml,U,E	
*Cola lateritia*	Amoreira, maria-cujá, moreira	Fd,O	
*Gossypium hirsutum*	Algodão, algodoeiro, algodoeiro-vulgar	U	●
*Hibiscus cannabinus*	Malva-brava	Fd,U	
*Hibiscus rosa-sinensis*	Cardeal, cardiais	Fr,Ml,O	
*Hibiscus sabdariffa*	Bissap	Fd	●
*Hibiscus surattensis*		O	
*Sida salviifolia*	Lol-branco, lôlo-preto	Fr,U	
*Thespesia populnea*	Bela-sombra	O	
**Meliaceae**			
*Azadirachta indica*	Primo-de-morôdjo	P,E	
*Khaya senegalensis*	Mogno	Ml,E	
*Melia azedarach*	Intendente, tendente, tindint, viúva	T,U,O,E	
*Trichilia emetica*	Mafureira, mafurra, mafurreira, mufurreira	Fd,O	
**Moraceae**			
*Artocarpus altilis*	Fruta-pão	Fd	
*Artocarpus heterophyllus*	Jaqueira	Fd	
*Artocarpus integer*	Jaqueira	Fd	
*Ficus benjamina*	Figueira-brava-da-índia	O	
*Ficus carica*	Figueira, figueira-de-portugal	Fd	●
*Ficus elastica*	Borracheira	O	
*Ficus leonensis*		O	
*Ficus lutea*	Lemba-lemba	O	
*Ficus religiosa*	Figueira-de-goa, figueira-da-índia	O	
*Ficus thonningii*		Fr,O	
*Morus nigra*	Amoreira, morreira	Fd	
**Moringaceae**			
*Moringa oleifera*	Acácia-blanco, acácia-branca, moringa	Fd,Fr,Mt,O	
**Musaceae**			
*Musa* × *paradisiaca*	Banana-pão, bananeira	Fd,Ml	●
**Myrtaceae**			
*Corymbia citriodora*		O,E	
*Eucalyptus camaldulensis* subsp. *camaldulensis*	Calipe, calipo, calipto, eucalipto	Ml,O,E	
*Eucalyptus globulus*	Calipe, calipo, calipto, eucalipto	P,O	
*Eucalyptus gomphocephala*	Calipe, calipo, calipto, eucalipto	E	
*Eucalyptus pruinosa*	Calipe, calipo, calipto, eucalipto	O	
*Eucalyptus tereticornis*	Calipe, calipo, calipto, eucalipto	E	
*Eucalyptus viminalis*	Calipe, calipo, calipto, eucalipto	O	
*Eugenia uniflora*	Pitangueira	Fd	
*Psidium cattleyanum*	Araçá, goiavinha	Fd	
*Psidium guajava*	Goiabeira	Fd,Fr	●
*Syzygium jambos*	Jamboeiro, jambre	Fd,O	
**Nyctaginaceae**			
*Bougainvillea glabra*	Bongavilia, buganvílea	O	
*Bougainvillea spectabilis*	Buganvil, buganvila, buganvílea, mungavi	Ml,O	
*Mirabilis jalapa*	Batata-de-burro, batata-de-porco, gasimi, jesimi, maravilhas	Fr,O	
**Olacaceae**			
*Ximenia americana*	Ameixieira, ameixeira-brava	Fd	●
**Oleaceae**			
*Jasminum officinale*		O	
*Jasminum sambac*	Jasmineiro	O	
*Olea europaea* subsp. *europaea*	Oliveira, oliveira-brava, zambujeiro, zambujo	O	
**Oxalidaceae**			
*Averrhoa bilimbi*		Fd	
*Oxalis debilis*		O	
*Oxalis latifolia*	Azedinha	O	
**Papaveraceae**			
*Argemone mexicana*	Cardo, cardo-santo	Ml,Mt	●
**Passifloraceae**			
*Passiflora edulis*	Maracujá-pequeno	Fd	
*Passiflora quadrangularis*	Maracujá-grande	Fd	
**Petiveriaceae**			
*Rivina humilis*	Uva-de-macaco	Mt	
**Phyllanthaceae**			
*Phyllanthus acidus*	Azedinha, groselheira, groselha	Fd,O	
**Phytolaccaceae**			
*Phytolacca americana*	Capa-rosa, uva-macaco	Fd	
*Phytolacca dioica*	Bela-sombra	O,E	
**Pinaceae**			
*Pinus canariensis*		Fu,E	
*Pinus halepensis*		Fu,E	
*Pinus pinaster*		Fu,E	
*Pinus radiata*		Fu,E	
**Plantaginaceae**			
*Antirrhinum majus*	Boca-de-lobo-pequena, boca-dilobo, mataquim	Ml	
*Cymbalaria muralis*		O	
*Plantago major*	Fedegosa, tanchagem, tantchas	Fr	
**Plumbaginaceae**			
*Plumbago zeylanica*	Fogo-da-serra, joelho-de-cabra, mato-gonçalves, pega-cabrito	O	
**Poaceae**			
*Arundo donax*	Caniço, cariço	U	
*Avena sativa*	Palha-de-trigo	Fr	
*Bambusa vulgaris*	Bambu-grande, carisso-da-guiné	U	
*Coix lacryma-jobi*		O	
*Cymbopogon citratus*	Belgata, capim-limão, chá-de-príncipe, chali, xali	Fd	
*Eragrostis tenella*		Fr	
*Paspalum vaginatum*		Fr,O	
*Saccharum officinarum*	Cana-de-açúcar, cana-doce-preta	Fd,Fr	●
*Setaria parviflora*	Balanco, gôgô, rabo-de-gato, rabo-de-raposa	Fr	
*Sorghum bicolor*	Bimberim, sorgo	Fd,Fr,Ml	●
*Sorghum halepense*	Achada-carreira, sololo	Fr,U	
*Stenotaphrum secundatum*		Fr,O	
*Zea mays*	Midjo, milho, milho-de-capa-preta	Fd,Fr,Ml	●
**Polygonaceae**			
*Antigonon leptopus*	Fátima, rosa-di-campo, rosa-di-fátima, trepadeira-de-fátima	Ml,O	
*Coccoloba uvifera*	Bela-sombra, mogno	O	
**Proteaceae**			
*Grevillea robusta*	Carvalho-prateado, grevilia	Ml,T,O,E	
**Rosaceae**			
*Cydonia oblonga*	Gamboeiro, marmeleiro	Fd	●
*Fragaria × ananassa*	Morangueiro	Fd	
*Malus domestica*	Macieira	Fd	●
*Prunus persica*	Pessegueiro	Fd,O	
*Pyrus communis*	Pereira, pereira-mansa	Fd	●
*Rhaphiolepis bibas*	Nespereira, nespereira-do-japão	Fd	●
*Rosa* × *centifolia*	Roseira	O	
*Rosa moschata*	Roseira	O	
*Rosa sempervirens*	Roseira	O	
**Rubiaceae**			
*Cinchona pubescens*	Quineira	O	
*Coffea arabica*	Cafeeiro, cafezeiro	Fd,S	●
*Mitracarpus hirtus*	Beitece, beio-teso, beiteso, locotém	Fr,P	
*Morinda citrifolia*	Noni	Fd	
*Spermacoce verticillata*	Bedjo-teso, biteso, lactane, locotane, locotano, velho-teso	Fr	
**Rutaceae**			
*Chloroxylon swietenia*	Pau-setim	O	
*Citrus* × *aurantium*	Laranjeira, laranjeira-azeda, laranjeira-doce	Fd,Ml	●
*Citrus* × *limon* var. *bergamia* [a]	Bergamo, bergamota	Fd	
*Citrus* × *limon* var. *limon*	Limoeiro	Fd,Ml	●
*Citrus maxima*	Toranjeira	Fd	
*Citrus medica*	Cidreira, limoeiro	Fd	●
*Citrus* x *aurantiifolia*	Limeira, limeira-azeda, limoeiro-pequeno	Fd	●
*Ruta chalepensis*	Arruda, aruda	Ml,S	
*Triphasia trifolia*		O	
**Salicaceae**			
*Salix* x *fragilis*		O	
**Sapindaceae**			
*Melicoccus bijugatus*		O	
*Sapindus saponaria*	Aveleira, aveloa, avelon, boa-madeira, saboeira, sapodilha	P,T,Mt	
**Sapotaceae**			
*Manilkara zapota*	Nispere	Fd	
**Scrophulariaceae**			
*Myoporum tenuifolium*	Pitosporum	O	
**Simmondsiaceae**			
*Simmondsia chinensis*	Jojoba	Mt	
**Solanaceae**			
*Alkekengi officinarum*		Fd	
*Capsicum annuum*	Malagueta, malagueta-arredondada, malaguetona, pimentão	Fd	
*Capsicum baccatum*	Pimento	Fd,O	
*Capsicum frutescens*	Malagueta, malagueta-pontiaguda, malaguetinha, piripiri	Fd,P	●
*Datura innoxia*	Barbiaca-preta, barbiaca-preta, barbidjaca, berbiaca, berbilhaca, burbilhaca, cardo-preto, padja-fede, palha-fede	Ml,O	
*Nicotiana glauca*	Chaluteiro, charroteira, charuteiro, tabaco-bravo, tabamqueira	O	
*Nicotiana tabacum*	Erba, erva-brava, erva-santa, tabaco	S,O	●
*Petunia axillaris*	Petunia	O	
*Physalis peruviana*	Capucha, caputcha, uva-caneca, uva-canela, uva-madeira	Fd	●
*Solanum betaceum*	Tomate-arbóreo	Fd	
*Solanum lycopersicum*	Camacho, tomate, tomateiro, tomatinho	Fd	
*Solanum melongena*	Beringela, beringelo, bringela	Fd	
*Solanum tuberosum*	Batata, batata-inglesa, batateira	Fd	
**Talinaceae**			
*Talinum paniculatum*	Laranjeirinha, limãozinho	Fd,O	
**Tamaricaceae**			
*Tamarix canariensis*	Tarafe, tarrafe, tamargueira	E	
**Tropaeolaceae**			
*Tropaeolum majus*	Chagas	O	
**Verbenaceae**			
*Aloysia citrodora*	Lúcia-lima	O	
*Lantana camara*	Freira, kambara, lantana-cor-de-rosa, lantuna, lantuna-amarela	Fr,O,E	
*Verbena officinalis*	Agibon-da-terra, gibon, gilbom, verbena	Fd	
*Verbena tweedieana*		O	
**Vitaceae**			
*Vitis vinifera*	Uveira, vinha	Fd	●
**Zingiberaceae**			
*Etlingera elatior*	Rosa-de-porcelana	O	
*Hedychium gardnerianum*		O	
*Zingiber officinale*	Gengibre	Fd	
**Zygophyllaceae**			
*Balanites aegyptiaca*		E	
*Tribulus cistoides*	Abreodjo, abre-olho, abriolha, abrochona, abroio, abrolho	Fr,Ml	

[a] According to Kalita et al. [35]. ● Taxa with reported historical use.

## Data Availability

We confirm that all data are original and provided in Tables and Figures within the article and in the Supplementary Materials.

## Supplementary Materials

The following supporting information can be downloaded at: https://www.mdpi.com/article/10.3390/plants11101313/s1. Table S1. Useful plant species in Cabo Verde islands: taxonomic diversity, origin and uses. Table S2. Geographic, demographic and economic indicators for Cabo Verde.
