# Supplementary material for: Diversity of Useful Plants in Cabo Verde Islands: A Biogeographic and Conservation Perspective
Source: Plants (Basel). 2022 May 15;11(10):1313. doi: 10.3390/plants11101313 (PMC9144021; doi:10.3390/plants11101313)
Supplement: Supplementary file 1 [file plants-11-01313-s001.zip › plants-1711739-supplementary.pdf]

plants

MDPI

Supplementary Data

Diversity of Useful Plants in Cabo Verde Islands: A Biogeographic and Conservation Perspective

Maria Cristina Duarte [1,*], Isildo Gomes [2], Silvia Catarino [3,4], Miguel Brilhante [3], Samuel Gomes [2], Aline Rendall [2], Ângela Moreno [2], Arlindo Rodrigues Fortes [5,6], Vladmir Silves Ferreira [5], Isaurinda Baptista [5], Herculano Dinis [7], and Maria Manuel Romeiras [1,3]

Table S1. Useful plant species in Cabo Verde islands: taxonomic diversity, origin and uses.

Cabo Verde	Cabo Verde	Santo Antão	São Vicente	São Nicolau	Sal	Boavista	Maio	Santiago	Fogo	Brava
Total flora										
Total taxa Nº (1)	813	521	299	349	147	219	232	498	409	262
Useful taxa										
Families Nº	88	83	51	63	41	45	45	76	65	59
Genera Nº	338	255	144	168	99	118	127	280	221	191
Taxa Nº	518	372	203	234	123	158	169	388	297	243
Exotic taxa Nº	373	268	117	147	77	95	95	269	199	169
Native taxa Nº	145	104	86	87	46	63	74	119	98	74
Native non-endemic taxa Nº	107	268	117	147	77	95	95	269	199	169
Endemic taxa Nº	38	22	20	20	9	9	6	23	21	17
Total uses Nº	757	573	338	391	224	280	283	611	482	401
Food Nº	158	124	71	75	48	65	62	131	104	90
Forage / pasture Nº	171	134	94	101	53	71	81	149	118	98
Melliferous Nº	71	63	42	49	29	38	36	70	58	54
Poison Nº	12	8	8	5	5	6	6	9	8	6
Social Nº	9	8	5	7	3	4	4	8	6	5
Fuelwood Nº	21	15	9	12	9	10	10	15	11	15
Timber Nº	9	8	6	7	4	5	4	9	8	4
Utilitarian Nº	30	22	16	19	9	12	13	28	19	19
Materials Nº	21	17	9	12	7	9	8	20	14	10
Ornamental Nº	183	118	50	64	36	37	39	123	94	66
Environmental Nº	72	56	28	40	21	23	20	49	42	34

(1) According to Sánchez-Pinto et al. [63].

Table S2. Geographic, demographic and economic indicators for Cabo Verde.

Indicators	Santo Antão	São Vicente	São Nicolau	Sal	Boavista	Maio	Santiago	Fogo	Brava
Area km² [(1)]	779	227	343	216	620	269	991	476	64
Altitude m [(1)]	1979	774	1304	406	378	436	1392	2829	976
Population Nº [(1)]	38194	84228	12108	39696	18792	7352	309638	35015	5463
Agriculture ha [(2)]	5036.6	569.4	999.4	73.5	275.4	388.6	20530.9	6731	518.9
Forest ha [(2)]	2177.8	2163.1	2464.1	356	1822.6	5446.9	37328.3	2347.4	813.2
Rural population Nº [(3)]	20904	6006	7857	2350	1657	3167	114486	22747	3222
Farms Nº [(3)]	5822	1701	2096	544	509	898	26841	6023	965
Rainfed farming Nº [(3)]	3648	227	1427	274	107	467	20803	5506	850
Irrigated farming Nº [(3)]	2203	301	542	15	65	103	5093	154	104
Livestock farming Nº [(3)]	4571	1445	1730	383	500	836	23752	4946	585
Forest holdings Nº [(3)]	54	47	5	0	0	147	11361	2645	117
Population using fuelwood Nº [(4)]	15810	3438	1731	0	233	2145	92049	17848	615
Tourists Nº (in 2019) [(5)]	35599	40074	902	353784	237416	981	76884	11468	874
Nominal GDP (in 2017, million ECV) [(1)]	10069	26876	3324	21744	9643	1550	89987	8634	1271

(1) Anuário Estatístico 2019 [14].
(2) Inventário Florestal Nacional de Cabo Verde 2012 [102].
(3) V Recenseamento Geral da Agricultura 2015 [101].
(4) Estatísticas do Ambiente 2016 [99].
(5) Avaliação de Impacto da Pandemia da COVID-19 [100].
